# Dopamine-induced pruning in monocyte-derived-neuronal-like cells (MDNCs) from patients with schizophrenia

**DOI:** 10.1038/s41380-022-01514-w

**Published:** 2022-04-01

**Authors:** Alfredo Bellon, Vincent Feuillet, Alonso Cortez-Resendiz, Faycal Mouaffak, Lan Kong, L. Elliot Hong, Lilian De Godoy, Therese M. Jay, Anne Hosmalin, Marie-Odile Krebs

**Affiliations:** 1 Department of Psychiatry and Behavioral Health, Penn State Hershey Medical Center, Hershey, PA USA; 2 Department of Pharmacology, Penn State Hershey Medical Center, Hershey, PA USA; 3 Aix-Marseille University, CNRS, INSERM, Centre d’Immunologie de Marseille-Luminy, Marseille, France; 4 Université de Paris, Institut Cochin, CNRS, INSERM, F-75014 Paris, France; 5 Institute of Psychiatry and Neuroscience of Paris (IPNP), INSERM U1266, Pathophysiology of Psychiatric Disorders, Université de Paris, Paris, France; 6 Pôle de Psychiatrie d’Adultes 93G04, EPS Ville Evrard, Saint Denis, France; 7 Department of Public Health Sciences, Penn State Hershey Medical Center, Hershey, PA USA; 8 Department of Psychiatry, Maryland Psychiatric Research Center, University of Maryland School of Medicine, Baltimore, MD USA; 9 Baptist Hospital, Miami, FL USA; 10 Groupe-Hospitalo-Universitaire de Paris, Psychiatrie et Neuroscience, Pôle PEPIT, University of Paris, Paris, France

**Keywords:** Schizophrenia, Neuroscience

## Abstract

The long lapse between the presumptive origin of schizophrenia (SCZ) during early development and its diagnosis in late adolescence has hindered the study of crucial neurodevelopmental processes directly in living patients. Dopamine, a neurotransmitter consistently associated with the pathophysiology of SCZ, participates in several aspects of brain development including pruning of neuronal extensions. Excessive pruning is considered the cause of the most consistent finding in SCZ, namely decreased brain volume. It is therefore possible that patients with SCZ carry an increased susceptibility to dopamine’s pruning effects and that this susceptibility would be more obvious in the early stages of neuronal development when dopamine pruning effects appear to be more prominent. Obtaining developing neurons from living patients is not feasible. Instead, we used Monocyte-Derived-Neuronal-like Cells (MDNCs) as these cells can be generated in only 20 days and deliver reproducible results. In this study, we expanded the number of individuals in whom we tested the reproducibility of MDNCs. We also deepened the characterization of MDNCs by comparing its neurostructure to that of human developing neurons. Moreover, we studied MDNCs from 12 controls and 13 patients with SCZ. Patients’ cells differentiate more efficiently, extend longer secondary neurites and grow more primary neurites. In addition, MDNCs from medicated patients expresses less D1R and prune more primary neurites when exposed to dopamine. Haloperidol did not influence our results but the role of other antipsychotics was not examined and thus, needs to be considered as a confounder.

## Introduction

Although schizophrenia (SCZ) is typically diagnosed in late adolescence or early adulthood, this illness is commonly considered as a neurodevelopmental disorder that originates from a combination of genetic predisposition and environmental factors [1]. This long lapse between SCZ presumptive origin and its diagnosis has hindered the study of early neurodevelopmental processes directly in living patients.

Not surprisingly, the neurodevelopmental process at fault in SCZ remains unidentified. However, evidence collected from adult patients provides some insights. The most consistent finding in SCZ is decreased brain volume [2, 3] which is attributed to a shrinkage in neuropil [4–6]. Neuropil reductions are evident across several brain regions [7–13]. These broad neurostructural abnormalities contradicts the current leading theory suggesting smaller brains in SCZ result from increased pruning of synapses exclusively in one brain region (the prefrontal cortex) and only during the transition between adolescence to adulthood [4, 6, 14, 15]. Also in conflict with such theory is evidence that the brains of patients are smaller before the clinical onset of SCZ [16–20] indicating the neuropil is compromised in earlier stages of development. In addition, synapses and spines are not the only affected components of the neuronal structure. Instead, total dendritic length is also reduced [21, 22] and the number of dendritic branches are likewise decreased [23, 24]. Consistent with widespread deficits in the neuronal structure, is the variety of neuronal types affected in SCZ, namely; pyramidal [21–24], GABAergic [25], dopaminergic [12] and Purkinje cells [11]. Further support of early and broad neurostructural deficits is that developing neurons generated from patients’ stem cells not yet committed into any particular neuronal type present shorter neuronal extensions [26, 27]. These widespread defects involving several components of the neuronal structure found in postmortem studies as well as in stem-cells-derived-developing-neurons, suggest that patients with SCZ may carry an increased susceptibility to pruning of neuronal extensions from early stages of neuronal development.

A second consistent pathophysiological aspect in SCZ, but rarely approached from a neurodevelopmental perspective, is its association with dopamine. This relationship relies on several lines of evidence. For instance, at high levels, dopamine produces psychotic symptoms in healthy individuals [28] and exacerbates psychosis in patients with SCZ [29, 30]. Accordingly, clinical efficacy of most antipsychotic medications is linked to blocking dopamine receptors [31, 32]. Neuroimaging studies also support the involvement of dopamine in SCZ. Positron emission tomography (PET) and single-photon emission computed tomography (SPECT) have consistently revealed increased presynaptic dopamine synthesis [33]. Even individuals at ultra-high risk for psychosis have shown elevated dopamine synthesis capacity [34]. Dopamine receptors 1 and 2 have also been associated with this illness. The first study to quantify dopamine D_1_ receptors (D1R) in living, unmedicated individuals with SCZ found it decreased [35]. Follow up results however, have been mixed [36, 37]. In contrast, reductions in D_2_ receptors (D2R) are commonly encountered [38]. Overall, the mechanisms of dopamine involvement in the pathophysiology of SCZ remains unsolved. A scarcely explored research avenue in SCZ, is the role of dopamine during early neurodevelopment. Dopamine participates in several aspects of brain development including sculpting the neuronal shape [39]. In vivo studies with animal models of neurodevelopment indicate that activation of D1R decreases neurite extensions [40–42]. In line with these results are cell culture experiments using developing neurons and neuronal cell lines in which dopamine elicits retraction of neuronal processes, mostly through activation of D1R [43–46].

Evidence of shorter neuronal extensions in patients with SCZ, together with the regularity in which dopamine has been associated with this psychotic disorder, and the role of dopamine in retracting neuronal extensions, compelled us to propose the following hypothesis: patients with SCZ carry an increased susceptibility to dopamine’s pruning effects. This susceptibility would be more obvious in the early stages of neuronal development when dopamine pruning effects appear to be more prominent. The main challenge to test this hypothesis is that obtaining developing neurons from living patients is not feasible. To circumvent this problem, we used Monocyte-Derived-Neuronal-like Cells (MDNCs).

We have recently developed a protocol to transdifferentiate human circulating monocytes into neuronal-like cells without the need for reprograming [47]. These MDNCs structurally resemble developing human neurons, conduct electrical activity and express several genes and proteins associated with the inherited predisposition to SCZ [47, 48]. There is also evidence to suggest that MDNCs form synapses. Indirect evidence comes from the numerous synaptic genes expressed by these cells [47, 48]. More compelling data originates from electrophysiological studies showing MDNCs conduct action potentials as well as excitatory postsynaptic potentials (EPSPs) and inhibitory postsynaptic potentials (IPSPs) [47]. In addition, MDNCs express D1R and retract its neuronal extensions when exposed to dopamine [47]. Moreover, MDNCs offer two advantages over other cellular models. First, the entire transdifferentiation process from somatic cell to neuronal-like cell takes only 20 days which contrasts with other models that take months to develop [47]. Second, we have previously shown that MDNCs deliver reproducible results with serial samples from the same individual [47]. Concerns have been raised about the reproducibility of results with other in vitro models [49, 50].

In this manuscript we expanded the number of individuals and serial samples in which we tested the reproducibility of MDNCs’ results. We then compared if the neurostructure of MDNCs behaves similarly to that of human developing neurons in vitro. The neurostructure of MDNCs from 12 controls and 13 patients with schizophrenia was thoroughly studied. Dopamine pruning effects on MDNCs were assessed, as well as the expression of D1R. The potential influence of haloperidol on our results was also examined.

## Materials and methods

### Subjects

All participants, after receiving full description of the study, gave their informed and written consent. All study procedures were approved by local ethics committees and were in accordance with the Helsinki declaration. Experiments pertaining to the cohort of patients and controls were approved by the ethics committee Ile de France II while experiments on the characterization of MDNCs involving only control individuals were approved by the Institutional Review Board at Penn State University (Study #00006911).

Patients were diagnosed using criteria described in the Diagnostic and Statistical Manual of Mental Disorders, 4th Edition (DSM-IV). Diagnosis was based on clinical interviews and clinical records. Patients were recruited from the Department of Psychiatry at Sainte-Anne Hospital in Paris, France. Healthy controls were recruited through local advertisements. Controls were screened to rule out any past or present history of DSM-IV axis 1 disorders. Only individuals older than 18 years old were recruited.

Thirty-five subjects, 16 controls and 19 patients with SCZ, were recruited for this study. In the SCZ group one patient was diagnosed with schizoaffective disorder and another with pervasive developmental disorder while one control had hemochromatosis (Table 1). One patient and one control provided blood samples in two separate occasions and were included as different subjects in the analysis. Eight individuals, five patients and 3 controls, were excluded from the study due to a mistake in the concentration of growth factors used during the transdifferentiation process. Of the 13 patients included in the analysis, two were not receiving any medications (Table 1). Some results from healthy subjects were included in a prior publication [47]. Additional information about the number, age range and gender of individuals included in each experiment can be found in Tables 2 and 3 as well as in Supplementary Table S1.

**Table 1:** Demographics and medication intake of individuals included in the analysis.

CONTROLS <*n* = 12, one tested twice>					SCHIZOPHRENIA <*n* = 13, one tested twice>				
Diagnosis	Age	Sex	Ethnicity	Medications (doses per day)	Diagnosis	Age	Sex	Ethnicity	Medications (doses per day)
N/A	19	M	Caucasian	None	Pervasive Developmental Disorder	19	M	Caucasian	Risperidone 6 mg, Valproic Acid 150 mg; Methylphenidate 20 mg
N/A	26	M	Caucasian	None	Schizophrenia Disorganized type	19	M^a^	Caucasian	Haloperidol decanote 150 mg/month, Zopiclone 7.5 mg, Lithium 800 mg, Chlorpromazine 25 mg, Cyamemazine 50 mg
N/A	27	M^a^	Caucasian	None	Schizophrenia Undifferentiated type	25	M	Caucasian	Clozapine 300 mg, Aripiprazole 15 mg
N/A	26	F	Caucasian	None	Schizophrenia Undifferentiated type	26	F	Caucasian	Aripiprazole 15 mg
N/A	30	M	Caucasian	None	Schizophrenia Undifferentiated type	29	M	Caucasian	Untreated
N/A	30	M	Caucasian	None	Schizophrenia Undifferentiated type	31	M	Middle Eastern	Clozapine 25 mg, Duloxetine 120 mg
N/A	30	F	Caucasian	None	Schizophrenia Paranoid type	44	F	Caucasian/Mixed	Risperidone 6 mg, Chlorpromazine 100 mg, Oxazepam 150 mg, Amimemazine 20 mg
N/A	33	M	Caucasian	None	Schizophrenia Disorganized type	34	M	Caucasian	Valproic Acid ER 1000 mg, Aripiprazole 15 mg, Escitalopram 10 mg
N/A	37	M	Caucasian	Loratadine 10mg	Schizophrenia Paranoid type	35	M	Caucasian	Untreated
Hemochromatosis	46	M	Unknown	None	Schizoaffective disorder	44	M	Caucasian	Clozapine 600 mg, Valproic Acid 1500 mg, Chlorpromazine 300 mg, Hydroxyzine 300 mg, Diazepam 15 mg
N/A	65	M	Caucasian	Clopidorgel 75mg, Previscan 20mg, Rampril 2.5mg, Simvastatin 40mg, Omeprazol 20mg	Schizophrenia Disorganized type	67	M	Caucasian	Amisulpride 100 mg
N/A	28	F	Caucasian	None	Schizophrenia Undifferentiated type	32	M	Caucasian	Aripiprazole 15 mg
					Schizophrenia Paranoid type	42	M	Caucasian	Haloperidol 30 mg, Zopiclone 7.5 mg, Chlorpromazine 600 mg, Diazepam 30 mg

^a^These individuals were tested twice.

**Table 2:** Demographics and number of samples from individuals recruited for experiments on reproducibility.

Subject	Gender	Age	Ethnicity	Days to 2nd sample	Days to 3rd sample
1	M	78	Caucasian	35	None
2	F	31	Caucasian	402	None
3	F	67	Caucasian	39	66
4	M	42	Caucasian	43	78
5	M	26	Caucasian	56	None
6	F	25*	Unknown	24	None
7	M	38	Asian	91	None
8	M	29	Caucasian	161	None

M = Male, F = Female, *estimated.

**Table 3:** Gender, age and number of monocytes and peripheral blood mononuclear cells at baseline from patients versus controls.

	Controls (*n* = 13)	Schizophrenia (*n* = 14)	*P* value
Sex	77% men	86% men	*P* = 0.64
Age (mean ± SEM) (range)	32.6 ± 3.2 (19–65 years)	33.2 ± 3.4 (19–67 years)	*P* = 0.88
Number of Monocytes (mean ± SEM) (range)	6.58 ± 0.72 (3.8–12.3 million)	8.02 ± 1.17 (2–17 million)	*P* = 0.30
Number of ^a^PBMCs (mean ± SEM) (range)	71.65 ± 6.06 (43–101.5 million)	59.28 ± 3.76 (39–88 million)	*P* = 0.09
Percentage of Monocytes (mean ± SEM) (range)	9.6 ± 0.64% (6.3–13.1%)	12.8 ± 1.3% (5–21.7%)	*P* = 0.04

^a^*PBMCs* Peripheral blood mononuclear cells.

### Cell culture

We followed our transdifferentiation protocol described in detail elsewhere [47] and in supplementary materials and methods. Pictures of cells were taken using a Nikon Eclipse Ti-S/L 100 inverted microscope equipped with a CoolSNAP Myo, 20 MHz, 2.8 Megapixel, 4.54 × 4.54 µm pixels camera and with a Nikon CFI Super fluor 20X DIC prism objective. Further information about the protocols we followed for collecting pictures and tracing cells can be found in supplementary materials and methods.

Treatments with colchicine (Sigma–Aldrich, C9754) and dopamine (Sigma-Aldrich, H8502-259), involved three concentrations for colchicine (0.4 µM, 0.5 µM and 0.75 µM) and two for dopamine (4 mM and 5 mM). Detail information about dopamine preparation as well as preincubations with a D1-like receptor antagonist is provided in supplementary materials and methods. Tracing and cell characterization were done blinded.

Human neurons were obtained from ScienCell Research laboratories (1520-10) and cultured following the manufacturer’s instructions. Further information about the procedures followed using human neurons is listed in Supplementary Materials and Methods.

### Flow cytometry

Flow cytometry was only used to measure levels of immunofluorescence for CD14 or to determine differences in the percentage of cells expressing nestin or D1R. The protocol we followed has been previously described [47] including incubation times with Trypspin-EDTA (0.05%), phenol red (ThermoFisher Scientific, 25300) of 4 min for MDNCs and 15 min for macrophages [47, 51, 52]. Additional information about the methodology we followed for flow cytometry can be found in Supplementary Materials and Methods.

### Statistical analysis

We conducted a descriptive analysis to examine the distributions of measures from MDNCs for healthy controls and SCZ patients. Subject-level measurements for MDNCs were obtained by first averaging the picture-level data for each sample and then averaging the sample-level data within each subject. Differentiation efficiency and structural parameters were summarized at the subject-level and compared between study groups using analysis of variance (ANOVA) or two-sample *t* tests. The data at picture- or sample-level were also analyzed with a mixed model analysis to account for correlations of repeated measures within subjects. To compare pruning effects of experimental conditions between study groups, we adjusted for baseline retraction (response under control conditions), differentiation efficiency, and structure at baseline because these factors may affect the pruning effects of each treatment condition (dopamine & colchicine). Since different samples were used to measure these factors, we were unable to perform analysis with picture level data. Therefore, we ran linear regression analysis based on subject-level averaged data adjusting for these factors in the models as covariates. Least square means and standard errors were estimated from the models for each study group. Other statistical tests used are described in supplementary materials and methods.

## Results

### Reproducibility of results with MDNCs

We have previously shown that the neuronal structure of MDNCs transdifferentiated from two serial blood samples from four healthy men were not statistically different [47]. Here we expand our cohort to eight healthy individuals, five men and three women (Table 2 & Supplementary Table S1). We also increased the number of samples in two subjects from two to three serial samples. For each parameter studied, we compared the distributions between samples within each subject using Kolmogorov-Smirnov Tests and construct 95% confidence intervals for sample means and mean differences between samples.

**Fig. 1:**
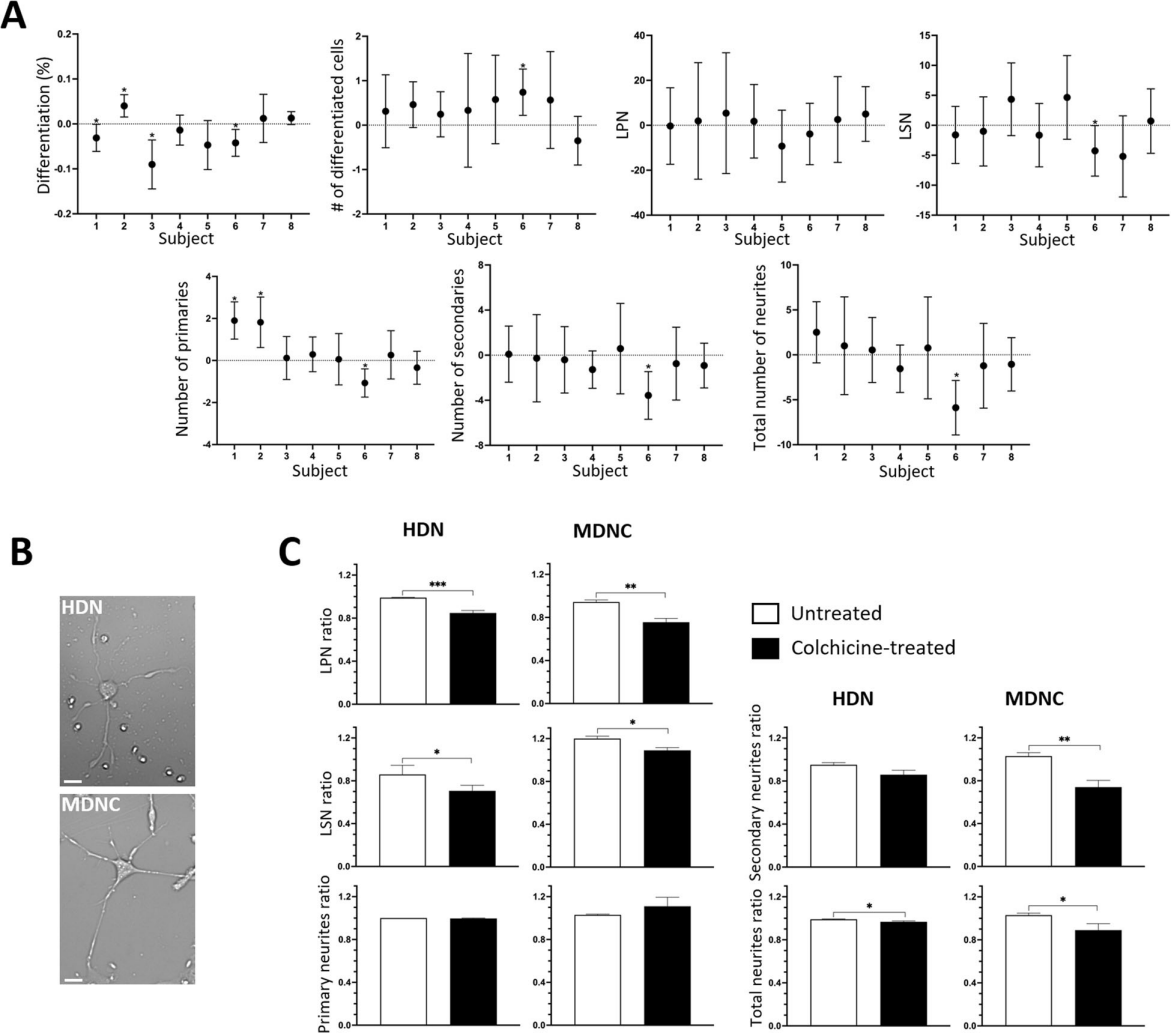
Reproducibility of results with MDNCs and structural comparison with human developing neurons. **A** Confidence interval plots in which dots represent means of differences between samples and error bars correspond to 95% confidence interval constructed using the Kolmogorov–Smirnov Test. The parameters presented are: differentiation percentage (subject 1, sample 1 (S1) *n* = 336 (cells), S2 *n* = 433; subject 2, S1 *n* = 284, S2 *n* = 523; subject 3, S1 *n* = 167, S2 *n* = 133, S3 *n* = 464; subject 4, S1 *n* = 556, S2 *n* = 434, S3 *n* = 255; subject 5, S1 *n* = 251, S2 *n* = 193; subject 6, S1 *n* = 797, S2 *n* = 413; subject 7, S1 *n* = 167, S2 *n* = 180; subject 8, S1 *n* = 1027, S2 *n* = 1531), number of differentiated cells (subject 1, S1 *n* = 27, S2 *n* = 45; subject 2, S1 *n* = 20, S2 *n* = 19; subject 3, S1 *n* = 16, S2 *n* = 23, S3 *n* = 38; subject 4, S1 *n* = 36, S2 *n* = 33, S3 *n* = 30; subject 5, S1 *n* = 25, S2 *n* = 25; subject 6, S1 *n* = 74, S2 *n* = 54; subject 7, S1 *n* = 28, S2 *n* = 28; subject 8, S1 *n* = 44, S2 *n* = 57), longest primary neurite (LPN) (subject 1, S1 *n* = 27, S2 *n* = 45; subject 2, S1 *n* = 20, S2 *n* = 19; subject 3, S1 *n* = 16, S2 *n* = 23, S3 *n* = 38; subject 4, S1 *n* = 36, S2 *n* = 33, S3 *n* = 30; subject 5, S1 *n* = 25, S2 *n* = 39; subject 6, S1 *n* = 74, S2 *n* = 54; subject 7, S1 *n* = 28, S2 *n* = 28; subject 8, S1 *n* = 44, S2 *n* = 57), longest secondary neurite (LSN) (subject 1, S1 *n* = 27, S2 *n* = 45; subject 2, S1 *n* = 20, S2 *n* = 19; subject 3, S1 *n* = 16, S2 *n* = 23, S3 *n* = 38; subject 4, S1 *n* = 35, S2 *n* = 33, S3 *n* = 30; subject 5, S1 *n* = 25, S2 *n* = 39; subject 6, S1 *n* = 67, S2 *n* = 54; subject 7, S1 *n* = 28, S2 *n* = 28; subject 8, S1 *n* = 42, S2 *n* = 56), number of primary neurites (subject 1, S1 *n* = 26, S2 *n* = 37; subject 2, S1 *n* = 12, S2 *n* = 13; subject 3, S1 *n* = 14, S2 *n* = 19, S3 *n* = 32; subject 4, S1 *n* = 22, S2 *n* = 25, S3 *n* = 23; subject 5, S1 *n* = 25, S2 *n* = 32; subject 6, S1 *n* = 51, S2 *n* = 45; subject 7, S1 *n* = 26, S2 *n* = 23; subject 8, S1 *n* = 38, S2 *n* = 48), number of secondary neurites and number of total neurites (same *n* for both) (subject 1, S1 *n* = 27, S2 *n* = 45; subject 2, S1 *n* = 20, S2 *n* = 19; subject 3, S1 *n* = 16, S2 *n* = 23, S3 *n* = 38; subject 4, S1 *n* = 36, S2 *n* = 33, S3 *n* = 30; subject 5, S1 *n* = 25, S2 *n* = 39; subject 6, S1 *n* = 74, S2 *n* = 54; subject 7, S1 *n* = 28, S2 *n* = 28; subject 8, S1 *n* = 44, S2 *n* = 57). *P = or < 0.05. **B** Light microscopy photographs of monocyte-derived-neuronal-like cells (MDNC) and human developing neurons (HDN) in culture for 5 days (20x original magnification). Scale bar = 20 µm. **C** Bar graphs showing the structural response to colchicine in HDNs and MDNCs. Data are expressed as ratios between the number of each structural parameter studied (LPN, LSN, number of primary and secondary neurites and total number of neurites) at baseline and after an hour of incubation either under control conditions or after treatment with colchicine 0.5 µM. Statistics are given as mean ± SEM. Differences were assessed using the Mann–Whitney test. Experiments for HDN come from 13 wells for each condition obtained from two different vials of human neurons. HDN for LPN, number of primary neurites and total number of neurites, control, *n* = 267 and colchicine, *n* = 261. For LSN control, *n* = 90 and colchicine, *n* = 125 and for number of secondary neurites control, *n* = 90 and colchicine, *n* = 123. Control MDNCs came from 8 donors and MDNCs treated with colchicine from 4 donors. MDNCs for LPN, number of primary neurites and total number of neurites, *n* = 656 control, *n* = 401 colchicine. MDNCs for LSN, *n* = 571 control, *n* = 267 colchicine. MDNCs for number of secondary neurites, *n* = 611 control, *n* = 357. *P = or < 0.05; **P = or < 0.008; ***P < 0.0001.

Differentiation percentage established by cellular phenotype yield significant differences between serial samples for subjects 1, 2, 3 and 6 but not for the other individuals, as shown in Fig. 1A and Supplementary Table S2. The number of differentiated cells evidence statistical significant differences between samples only for subject 6. No differences were observed in all other individuals (Fig. 1A & Supplementary Table S3).

Different neurostructural parameters of MDNCs were assessed: longest primary neurite (LPN), longest secondary neurite (LSN), number of primary neurites, number of secondary neurites, and number of all neurites (including primary, secondary, tertiary and quaternary). There are no statistical differences in LPN in any of the 8 subjects (Fig. 1A & Supplementary Table S4). For LSN, only subject 6 exhibits statistical changes between samples whereas all other participants do not (Fig. 1A & Supplementary Table S5). Number of primary neurites reveals differences in subjects 1, 2 and 6 but not in subjects 3, 4, 5, 7 & 8 (Fig. 1A & Supplementary Table S6). Number of secondary neurites reveals differences only for subject 6 whereas no statistical changes are evident in the rest of the cohort (Fig. 1A & Supplementary Table S7). Statistical differences for total number of neurites were limited to subject 6. All serial samples from the other 7 participants evidence no statistical change (Fig. 1A & Supplementary Table S8).

### Human developing neurons versus MDNCs

In a previous publication we showed that the structure of MDNCs is comparable to the structure of Human Developing Neurons (HDNs) after 5 days in culture [47] (Fig. 1B). Here we compare how HDNs and MDNCs from healthy controls (Supplementary Table S1) respond to colchicine 0.5 µM. Colchicine was chosen because it is well-known within the neuroscientific field for its ability to elicit pruning of neuronal extensions [53]. In addition, the mechanism of action of colchicine is well-understood and it is independent of membrane receptors as it acts by directly depolymerizing microtubules [54].

Under controlled conditions both HDNs and MDNCs retract their LPN slightly after one hour of incubation. Treatment with colchicine 0.5 µM for one hour leads to additional retraction of LPN that is comparable in both cell types. HDNs present a net LPN retraction of 14%, which is significantly different when compared to the level of retraction under control conditions. Likewise, MDNCs present an LPN net retraction of 19% when incubated with colchicine (Fig. 1C & Table 4). The behavior of secondary neurites from HDNs and MDNCs differs. Under control conditions, HDNs retract their LSN by 15% while MDNCs’ LSN grow by 20%. Exposure to colchicine 0.5 µM elicits even further retraction of HDNs’ LSN by another 15% whereas for MCNCs, colchicine prevents growth of LSN by 11%.

**Table 4:** Structural comparison between Human Developing Neurons (HDNs) and Monocyte-derived-neuronal-like cells (MDNCs) after treatment with colchicine.

Structural Parameter	HDNs^a^		*P* value	MDNCs^a^		*P* value
	Control	Colchicine		Control	Colchicine	
LPN	0.99 ± 0.004	0.848 ± 0.023	<0.00001	0.943 ± 0.019	0.756 ± 0.035	0.0067
LSN	0.859 ± 0.086	0.706 ± 0.053	0.05	1.2 ± 0.022	1.09 ± 0.026	0.019
Primary Neurites	1 ± 0.00	0.997 ± 0.003	0.75	1.03 ± 0.006	1.11 ± 0.084	0.88
Secondary Neurites	0.951 ± 0.021	0.859 ± 0.042	0.12	1.03 ± 0.032	0.74 ± 0.064	0.008
All Neurites	0.99 ± 0.004	0.96 ± 0.008	0.04	1.03 ± 0.019	0.89 ± 0.06	0.03

^a^Data are expressed as ratios.

*LPN* longest primary neurite, *LSN* longest secondary neurite.

Pruning of primary neurites is comparable between HDNs and MDNCs. Neither HDNs nor MDNCs prune any of its primary neurites after one hour of culture under control conditions. Treatment with colchicine leads to no statistical significant change in the number of primary neurites for either HDNs or MDNCs. Pruning of secondary neurites is different between HDNs and MDNCs. Control conditions cause minimal pruning of secondary neurites in HDNs, while MDNCs do not lose secondary neurites. Incubation with colchicine eliminates an additional 10% in the number of secondary neurites for HDNs but this difference is not statistically significant. For MDNCs, colchicine elicits a 25% reduction in the number of secondary neurites and this effect reaches statistical significance. When all neurites are included in the analysis, HDNs and MDNCs present comparable results. No changes are seen under control conditions for both cell types. Treatment with colchicine however, leads to a statistically significant reduction in the total number of neurites for HDNs as well as for MDNCs (Fig. 1C & Table 4).

### Monocytes from controls versus SCZ at baseline and during early stages of transdifferentiation

Twenty-five individuals, 12 controls (CTL) and 12 patients with SCZ plus one patient with pervasive developmental disorder and psychosis were included in the analysis. Two patients with SCZ were not taking any medications. The other 11 patients were receiving antipsychotics as well as other psychotropic medications (Table 1). Two-sample t-tests assuming unequal variances were used to compare mean differences between groups on continuous variables and Fisher’s exact tests were used for categorical variables. No statistical differences between groups are evidenced in gender, age, total number of monocytes per blood sample or, total number of peripheral blood mononuclear cells (PBMCs) per blood sample. The percentage of monocytes within PBMCs presents a small but statically significant difference between groups (Table 3). We ran the same analysis for gender, age, number of monocytes and PBMCs as well as percentage of monocytes excluding the patient with pervasive developmental disorder and our results remained the same (Supplementary Table S9). For one control we did not have access to the number of PBMCs or monocytes.

**Fig. 2:**
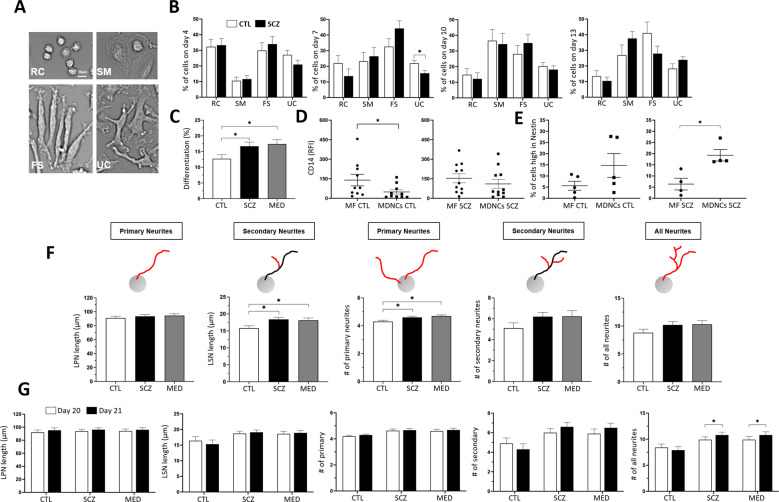
MDNCs from controls (CTL) versus patients with schizophrenia (SCZ). **A** Representative light microscopy photographs of the four different morphologies characterized during transdifferentiation: rounded cells (RC), standard macrophages (SM), fibroblastic shape (FS) and uncharacterized (UC) (20x original magnification). Scale bar = 20 µm. **B** Bar graphs showing variations between controls and patients with schizophrenia in the percentage of each of the four cell morphologies on day 4, 7, 10 and 13 of transdifferentiation. Repeated ANOVAs based on the mixed model framework were performed to examine group differences for each day. Data are given as mean ± SEM. Cells from 12 CTL and 13 SCZ were characterized for day 4, 7 & 10 and 5 CTL and 11 SCZ for day 13. Cells characterized on day 4, CTL *n* = 8437, SCZ *n* = 8204; on day 7, CTL *n* = 7783, SCZ *n* = 7609; on day 10, CTL *n* = 7125, SCZ *n* = 6830; and on day 13, CTL *n* = 2475, SCZ *n* = 5342. *P = or < 0.05. **C** Bar graphs contrasting the percentage of differentiated cells between CTL, SCZ and only medicated patients (MED). CTL, 12.7 ± 1.3%; SCZ, 16.7 ± 1.3%; *P* = 0.04; MED, 17.4 ± 1.4%; *P* = 0.027. Differentiation was obtained via cell phenotype. To determine differences between groups, multilevel mixed models to account for correlations of repeated measures at subject level and sample level were used, followed by a two-sample *t* test. Data are given as mean ± SEM. Cells from 12 CTL and 13 SCZ (of which 11 are medicated “MED”) were included in the analysis. Characterized cells for CTL, *n* = 32791; SCZ *n* = 37281; and for MED *n* = 32986. *P = or < 0.05, **P < 0.03. **D** Dot plots contrasting relative fluorescence intensity (RFI) of CD14 in macrophages (MF) versus MDNCs from either CTL or SCZ. CD14 RFI was measured via flow cytometry. For CTL, MF, 140 ± 44; MDNCs, 49 ± 17; *P* = 0.03; for SCZ, MF, 154 ± 34.5; MDNCs, 110 ± 33.8; *P* = 0.25. Differences were assessed using the Mann–Whitney test. Data are given as mean ± SEM. Cells from 10 CTL and 11 SCZ were included in the analysis. *P = or < 0.05. **E** Dot plots contrasting the percentage of cells expressing high levels of nestin in MF versus MDNCs from either CTL or SCZ. Nestin expression was measured via flow cytometry. For CTL, MF, 5.6 ± 2%; MDNCs, 14.7 ± 5.3%; *P* = 0.4 and for SCZ, MF, 6.3 ± 2.6%; MDNCs, 19.2 ± 2.5%; *P* = 0.01. Differences were assessed using the Mann–Whitney test. Data are given as mean ± SEM. Cells from 2 CTL and 4 SCZ were included in the analysis. One CTL was tested twice by duplicate. *P = or < 0.05. **F** Bar graphs contrasting several structural parameters at baseline between CTL, SCZ and MED. Structural parameters include: Longest primary neurite (LPN) (CTL, 91 ± 2.6 µm; SCZ, 93.5 ± 2.4 µm; *P* = 0.49, MED, 94.5 ± 2.6 µm; *P* = 0.36); longest secondary neurite (LSN) (CTL, 15.8 ± 0.7 µm; SCZ, 18.4 ± 0.6 µm; *P* = 0.02, MED, 18.1 ± 0.7 µm; *P* = 0.04); number of primary neurites (CTL, 4.3 ± 0.09; SCZ, 4.6 ± 0.08; *P* = 0.04, MED, 4.6 ± 0.09; *P* = 0.01); number of secondary neurites (CTL, 5.1 ± 0.5; SCZ, 6.2 ± 0.4; *P* = 0.17, MED, 6.2 ± 0.5; *P* = 0.19) and total number of neurites (CTL, 8.8 ± 0.6; SCZ, 10.2 ± 0.6; *P* = 0.11, MED, 10.3 ± 0.6; *P* = 0.13). To determine differences between groups, multilevel mixed models to account for correlations of repeated measures at subject level and sample level were used, followed by a two-sample *t* test. Data are given as mean ± SEM. MDNCs from 12 CTL and 13 SCZ (of which 11 are medicated “MED”) were included in the analysis. MDNCs traced for CTL *n* = 3933; SCZ *n* = 6144; and MED *n* = 5601. *P = or < 0.05, **P < 0.03. **G** Bar graphs contrasting several structural parameters on day 20 versus day 21 from CTL, SCZ and MED. Structural parameters studied were; longest primary neurite (LPN), longest secondary neurite (LSN), number of primary neurites, number of secondary neurites and total number of neurites. Two-sample t-tests was used to determine differences between CTL vs. SCZ and CTL vs. MED after a mixed model analysis was performed to account for correlations of repeated measures within subjects. Data are given as mean ± SEM. MDNCs from 7 CTL and 11 SCZ (of which 10 are medicated “MED”) were included in the analysis. MDNCs traced for LSN for CTL, day 20 *n* = 1090 and day 21 *n* = 1091; for all other structural parameters; CTL, day 20 *n* = 1189 and day 21 *n* = 1213; for LSN for SCZ, day 20 *n* = 2062 and day 21 *n* = 2152; for all other structural parameters day 20 *n* = 2193 and day 21 *n* = 2254; for LSN for MED, day 20 *n* = 1985 and day 21 *n* = 2018; for all other structural parameters day 20 *n* = 2114 and day 21 *n* = 2118. *P = or < 0.05, **P < 0.03.

After isolating monocytes from blood samples, we followed our 20-day transdifferentiation protocol [47]. As previously described, monocytes undergo four structural stages before reaching transdifferentiation into neuronal-like cells [47], including: (1) rounded cells (RC); (2) standard macrophages (SM), (3) fibroblastic shape (FS) and (4) uncharacterized cells (UC), which include cells that do not fit into any of the other 3 descriptions (Fig. 2A). These structural stages were quantified through microphotographs taken from one cell culture well on days when media was changed, namely; days 4, 7, 10 and 13.

Means and standard deviations were summarized at subject level by study groups. Repeated ANOVAs based on the mixed model framework were performed to examine group differences for each day. Random effect was specified in the models to account for correlations between microphotographs within each subject or variation among subjects. With this approach, the structural path to transdifferentiation between patients and controls shows that there are no statistical differences on days 4, 10 and 13 on any of the structural stages. On day 7, patients evidence less uncharacterized cells while the rest of the structural stages do not differ from controls (Fig. 2B & Supplementary Table S10). Analysis of these data excluding a patient with pervasive developmental disorder did not change our results (Supplementary Table S11).

### MDNCs from CTL versus SCZ

After twenty days in culture following our transdifferentiation protocol, around 13% of monocytes acquire a neuronal morphology characterized by the presence of a well-defined soma and long thin neurites [47] (Fig. 1B). We have previously shown that these Monocyte-Derived-Neuronal-like Cells (MDNCs) express a variety of neuronal markers [47, 48] and conduct electrical activity [47]. In order to compare the percentage of differentiated cells between groups, we collected data through microphotographs taken from multiple samples of each subject. We used multilevel mixed models to account for correlations of repeated measures at subject level and sample level, followed by a two-sample *t* test. This approach reveals that the percentage of cells that acquire a neuronal morphology is higher in patients with SCZ than in CTL. Such statistical difference is still present when only medicated patients (MED) are included in the analysis (Fig. 2C). If a patient with pervasive developmental disorder is excluded from the analysis a statistical trend towards higher differentiation in patients remains while, a statistical significant difference is still evident when this patient is excluded from the cohort of only medicated patients (CTL, 12.7 ± 1.3%; SCZ, 16.4 ± 1.9%; *P* = 0.07; MED, 17.1 ± 2.0%; *P* = 0.04).

In a prior publication we showed that MDNCs reduce or even completely abolish its expression of CD14 [47], a surface marker for monocytes and macrophages. It is therefore expected that MDNCs will express significantly lower CD14 if compared with macrophages from the same individual. Relative fluorescence intensity (RFI) obtained via flow cytometry as an indicator of CD14 expression shows MDNCs from CTL behave as predicted. In contrast, MDNCs from SCZ do not (Fig. 2D). Expression of nestin is also different between groups based on flow cytometry results. The percentage of MDNCs expressing high levels of nestin is higher than in macrophages in both cohorts but for CTL this difference is not statistically significant (Fig. 2E). Likewise, the neurostructure of MDNCs reveals differences. MDNCs from patients with SCZ are structurally more complex than cells from CTL. While no differences are evident when comparing LPN between CTL and either SCZ or MED, MDNCs from patients extend longer secondary neurites and grow more primary neurites and the same is true for MED (Fig. 2F). The number of secondary neurites as well as the number of all neurites does not show statistical differences between groups. When a patient with pervasive developmental disorder is excluded from the analysis, the only difference is a trend to significance in the length of LSN in medicated patients (Supplementary Tables S12 & S13). The rest of our results remained unchanged.

Since transdifferentiation occurs after 20 days in culture, the vast majority of MDNCs structural analyses were conducted between days 20 and 21. We analyzed whether there are structural differences between MDNCs at day 20 (D20) versus day 21 (D21). Only those individuals in each group for whom we have microphotographs of MDNCs at D20 and D21 were included in the analysis (Supplementary Table S1). MDNCs from controls do not show structural changes in any of the parameters studied namely; LPN, LSN, number of primary neurites, number of secondary neurites and total number of neurites (Supplementary Table S14). In contrast, MDNCs from SCZ evidence a higher number of total neurites on D21 when compared to D20, while the rest of the structural parameters present no statistical changes (Fig. 2G & Supplementary Table S15). When only medicated patients are included in the analysis, the number of total neurites on D21 is also higher than on D20. The rest of the structural parameter are unchanged. If a patient with pervasive developmental disorder is excluded from the analysis, there is a trend towards significance in the number of total neurites in patients with SCZ but there is no statistical difference when only medicated patients are analyzed (Supplementary Table S16).

### Structural responses to colchicine and dopamine in MDNCs from CTL versus SCZ

We select colchicine because we have previously shown that colchicine prunes neuronal extensions from MDNCs [47] comparably to neurons [55] and neuronal cell lines [56]. Moreover, colchicine acts independently of any membrane receptors and instead elicits microtubule depolymerization directly [54]. Likewise, dopamine prunes neuronal processes in neurons during early development [43, 44, 57] and we have found a similar response in MDNCs [47].

**Fig. 3:**
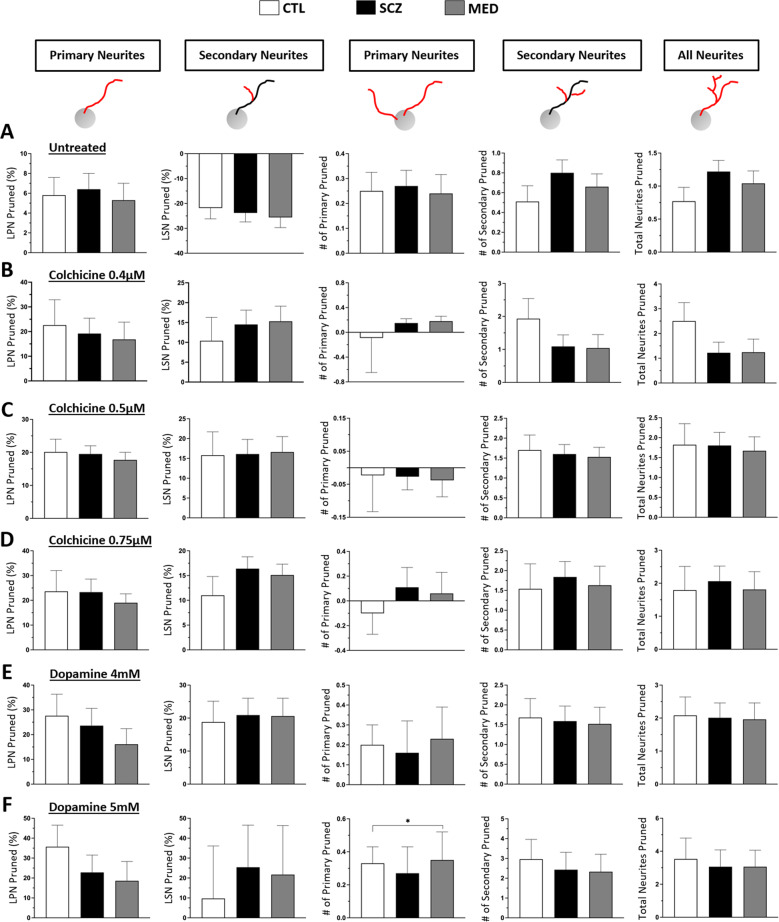
Structural responses to colchicine and dopamine in MDNCs from CTL versus SCZ. **A** Bar graphs contrasting the amount of pruning evidenced in MDNCs after an hour of culture under control conditions in cells from CTL, SCZ and MED. Structural parameters studied were; longest primary neurite (LPN), longest secondary neurite (LSN), number of primary neurites, number of secondary neurites and total number of neurites. Two-sample *t* tests was used to determine differences between CTL vs. SCZ and CTL vs. MED after a mixed model analysis was performed to account for correlations of repeated measures within subjects. Data are given as mean ± SEM. MDNCs from 8 CTL and 10 SCZ (of which 8 are medicated “MED”) were included in the analysis. MDNCs traced for LSN for CTL, *n* = 571; SCZ, *n* = 854; and MED, *n* = 667; for all other structural parameters; CTL, *n* = 656; SCZ, *n* = 976; and MED, *n* = 769. **B** Bar graphs contrasting the amount of pruning evidenced in MDNCs after an hour of incubation with colchicine 0.4 µM in cells from CTL, SCZ and MED. The same structural parameters as in (**A**) were studied. We performed linear regression analysis adjusting for baseline retraction (response under control conditions), differentiation efficiency, and structure at baseline in the models as covariates based on subject-level averaged data. Data are given as mean ± SEM. MDNCs from 3 CTL and 7 SCZ (of which 6 are medicated “MED”) were included in the analysis. MDNCs traced for LSN for CTL, *n* = 117; SCZ, *n* = 341; and MED, *n* = 327; for all other structural parameters; CTL, *n* = 191; SCZ, *n* = 405; and MED, *n* = 388. **C** Bar graphs contrasting the amount of pruning evidenced in MDNCs after an hour of incubation with colchicine 0.5 µM in cells from CTL, SCZ and MED. The same structural parameters and the same statistical analysis as in (**A**) were used. Data are given as mean ± SEM. MDNCs from 4 CTL and 9 SCZ (of which 8 are medicated “MED”) were included in the analysis. MDNCs traced for LSN for CTL, *n* = 267; SCZ, *n* = 565; and MED, *n* = 534; for all other structural parameters; CTL, *n* = 401; SCZ, *n* = 662; and MED, *n* = 627. **D** Bar graphs contrasting the amount of pruning evidenced in MDNCs after an hour of incubation with colchicine 0.75 µM in cells from CTL, SCZ and MED. The same structural parameters and the same statistical analysis as in (**A**) were used. Data are given as mean ± SEM. MDNCs from 3 CTL and 7 SCZ (of which 6 are medicated “MED”) were included in the analysis. MDNCs traced for LSN for CTL, *n* = 221; SCZ, *n* = 490; and MED, *n* = 472; for all other structural parameters; CTL, *n* = 297; SCZ, *n* = 621; and MED, *n* = 593. **E** Bar graphs contrasting the amount of pruning evidenced in MDNCs after an hour of incubation with dopamine 4mM in cells from CTL, SCZ and MED. The same structural parameters and the same statistical analysis as in (**A**) were used. Data are given as mean ± SEM. MDNCs from 6 CTL and 9 SCZ (of which 7 are medicated “MED”) were included in the analysis. MDNCs traced for LSN for CTL, *n* = 354; SCZ, *n* = 455; and MED, *n* = 414; for all other structural parameters; CTL, *n* = 477; SCZ, *n* = 622; and MED, *n* = 486. **F** Bar graphs contrasting the amount of pruning evidenced in MDNCs after an hour of incubation with dopamine 5 mM in cells from CTL, SCZ and MED. The same structural parameters and the same statistical analysis as in (**A**) were used. Data are given as mean ± SEM. MDNCs from 5 CTL and 7 SCZ (of which 6 are medicated “MED”) were included in the analysis. MDNCs traced for LSN for CTL, *n* = 228; SCZ, *n* = 316; and MED, *n* = 291; for all other structural parameters; CTL, *n* = 344; SCZ, *n* = 388; and MED, *n* = 352. *P = or < 0.05.

MDNCs from CTL and SCZ (Supplementary Table 1S) were cultured in parallel for one hour with three concentrations of colchicine, two concentrations of dopamine and under controls conditions. For control conditions, all subjects had one sample except for one who had two samples. Correlations between microphotographs within each subject was accounted for by random effect in the mixed models. MDNCs cultured under control conditions from all groups slightly retract their LPN whereas, LSN grows. Neither LPN nor LSN show any differences between groups. Such culture conditions also elicit pruning of a small number of primary, secondary and total number of neurites independently of diagnosis or medications (Fig. 3A & Table 5).

**Table 5:** Structural responses to colchicine and dopamine in MDNCs from control individuals (CTL) versus patients with schizophrenia (SCZ).

Treatment	Structural Component Pruned	CTL	SCZ	*P* value	MED^a^	*P* value
Control conditions	LPN (%)	5.8 ± 1.8	6.4 ± 1.6	0.81	5.3 ± 1.7	0.82
	LSN (%)	−21.8 ± 4.4	−23.8 ± 3.6	0.73	−25.6 ± 4.1	0.55
	# of Primaries	0.25 ± 0.07	0.27 ± 0.06	0.47	0.24 ± 0.07	0.57
	# of Secondaries	0.51 ± 0.16	0.8 ± 0.13	0.19	0.66 ± 0.13	0.47
	# of all neurites	0.77 ± 0.21	1.22 ± 0.17	0.12	1.04 ± 0.19	0.35
Colchicine 0.4 µM	LPN (%)	22.6 ± 10.3	19.2 ± 6.2	0.80	16.8 ± 7.0	0.69
	LSN (%)	10.4 ± 5.9	14.5 ± 3.6	0.60	15.3 ± 3.8	0.51
	# of Primaries	−0.08 ± 0.56	0.15 ± 0.07	0.27	0.18 ± 0.08	0.10
	# of Secondaries	1.93 ± 0.61	1.09 ± 0.65	0.33	1.04 ± 0.41	0.33
	# of all neurites	2.5 ± 0.74	1.2 ± 0.43	0.23	1.24 ± 0.53	0.30
Colchicine 0.5 µM	LPN (%)	20.1 ± 3.9	19.5 ± 2.5	0.90	17.7 ± 2.3	0.67
	LSN (%)	15.8 ± 5.9	16.1 ± 3.7	0.97	16.6 ± 3.9	0.89
	# of Primaries	0.023 ± 0.11	−0.027 ± 0.04	0.59	−0.038 ± 0.05	0.33
	# of Secondaries	1.7 ± 0.38	1.6 ± 0.24	0.24	1.53 ± 0.24	0.63
	# of all neurites	1.82 ± 0.53	1.80 ± 0.33	0.87	1.67 ± 0.35	0.80
Colchicine 0.75 µM	LPN (%)	23.6 ± 8.4	23.3 ± 5.3	0.97	19.0 ± 3.6	0.58
	LSN (%)	11.0 ± 3.8	16.4 ± 2.4	0.30	15.1 ± 2.2	0.37
	# of Primaries	−0.1 ± 0.17	0.11 ± 0.16	0.71	0.06 ± 0.17	0.81
	# of Secondaries	1.5 ± 0.63	1.8 ± 0.39	0.72	1.63 ± 0.48	0.83
	# of all neurites	1.7 ± 0.72	2.0 ± 0.46	0.77	1.81 ± 0.54	0.83
Dopamine 4 mM	LPN (%)	27.6 ± 8.7	23.6 ± 7.0	0.73	16.1 ± 6.3	0.23
	LSN (%)	18.8 ± 6.3	20.9 ± 5.1	0.81	20.6 ± 5.4	0.93
	# of Primaries	0.20 ± 0.10	0.16 ± 0.16	0.51	0.23 ± 0.16	0.51
	# of Secondaries	1.6 ± 0.48	1.59 ± 0.38	0.89	1.52 ± 0.42	0.72
	# of all neurites	2.08 ± 0.56	2.01 ± 0.45	0.93	1.96 ± 0.5	0.71
Dopamine 5 mM	LPN (%)	35.7 ± 10.8	22.8 ± 8.7	0.43	18.6 ± 9.7	0.31
	LSN (%)	9.7 ± 26.4	25.4 ± 21.1	0.69	21.7 ± 24.6	0.78
	# of Primaries	0.33 ± 0.10	0.27 ± 0.16	0.52	0.35 ± 0.17	0.05
	# of Secondaries	2.96 ± 1.0	2.43 ± 0.88	0.74	2.33 ± 0.88	0.62
	# of all neurites	3.53 ± 1.27	3.06 ± 1.02	0.80	3.06 ± 1.0	0.72

^a^Comparing only medicated patients (MED) versus CTL.

*LPN* longest primary neurite, *LSN* longest secondary neurite.

To compare pruning effects of colchicine and dopamine between groups, we adjusted for baseline retraction (response under control conditions), differentiation efficiency, and structure at baseline since these factors may affect the pruning effects of each treatment condition. After accounting for these variables, we found that treatment with colchicine 0.4 µM retracts LPN and LSN in all groups (Fig. 3B & Table 5). In contrast, colchicine does not change the number of primary neurites in any group. Secondary neurites and total number of neurites follow a similar pattern of pruning in all cohorts. Exposure to colchicine 0.5 µM generates results comparable to those observed with colchicine 0.4 µM. Colchicine at this intermediate concentration retracts LPN and LSN equally in every cohort (Fig. 3C & Table 5). In close resemblance to colchicine 0.4 µM, colchicine 0.5 µM elicits minimal changes in primary neurites, while the three groups retract a similar number of secondary neurites and total number of neurites. Results from the highest concentration of colchicine tested (0.75 µM) follow a similar pattern. Colchicine 0.75 µM reduces the size of LPN and LSN comparably in all cohorts, while the number of primary neurites remains stable. Secondary neurites and total number of neurites retract at equivalent rates independently of diagnosis or medications (Fig. 3D & Table 5).

MDNCs from the three groups incubated with dopamine 4 mM retract its LPN and LSN comparably. Likewise, pruning of primary, secondary and total number of neurites reveals no statistical differences (Fig. 3E & Table 5). There are also no statistical differences in the reduction of LPN or LSN after treatment with dopamine 5mM. In contrast, this higher concentration of dopamine prunes a slightly higher but statistically significant number of primary neurites in MDNCs from MED but not from SCZ when compared with CTL (Fig. 3F & Table 5). No significant differences are evident in the loss of secondary or total number of neurites.

### Dopamine 1 receptors in MDNCs from controls versus SCZ

**Fig. 4:**
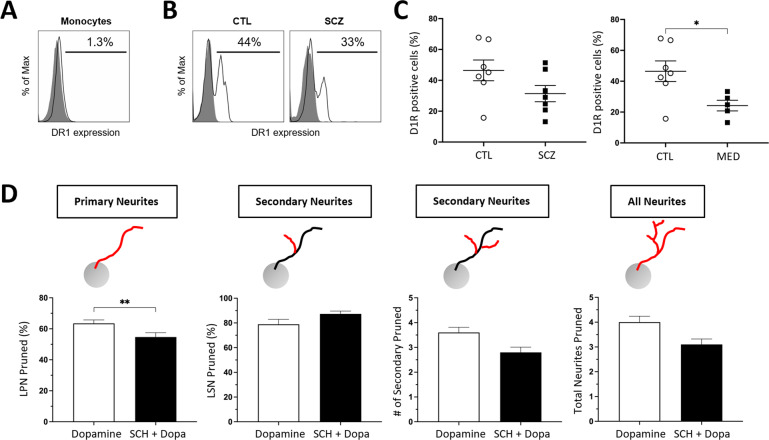
Dopamine 1 receptors in MDNCs from CTL and SCZ. **A** Flow cytometric diagram showing expression of dopamine 1 receptors (D1R) in monocytes. Gray histograms represent control isotypic labeling. White histograms represent specific labeling. **B** Flow cytometric diagrams showing expression of D1R in MDNCs from a healthy control and a patient with SCZ. Gray histograms represent control isotypic labeling. White histograms represent specific labeling. **C** Dot plots contrasting expression of D1R in MDNCs from CTL vs. SCZ (CTL, 46.4 ± 6.7; SCZ, 31.3 ± 5.2; *P* = 0.15) and CTL vs. MED (CTL, 46.4 ± 6.7; MED, 24.2 ± 3.4; *P* = 0.03). D1R expression was measured via flow cytometry. Non-parametric Mann–Whitney test was used to make pairwise comparisons between groups. Data are given as mean ± SEM. Cells from 7 CTL, 7 SCZ and 5 MED were included in the analysis. *P = or < 0.05. **D** Bar graphs showing the effects of SCH-23390, a D1R antagonist, in dopamine-elicited pruning in MDNCs. Dopamine was used at 4 mM. For LPN, dopamine, 63.5 ± 2.2%; SCH, 54.7 ± 2.8%; *P* = 0.0093; LSN, dopamine, 78.9 ± 4.0%; SCH, 87.3 ± 2.3%; *P* = 0.93; number of secondary neurites, dopamine, 3.6 ± 0.21; SCH, 2.8 ± 0.21; *P* = 0.08 and total number of neurites, dopamine, 4.0 ± 0.24; SCH, 3.1 ± 0.22; *P* = 0.08. The non-parametric Mann–Whitney test was used to make pairwise comparisons between groups. Data are given as mean ± SEM. MDNCs from three individuals were included in the analysis. One individual was an unmedicated patient and one healthy subject was tested by duplicate. MDNCs traced for LSN treated with dopamine *n* = 200; and with SCH-23390 + dopamine, *n* = 117; for all other structural parameters; treated with dopamine *n* = 221; and with SCH-23390 + dopamine, *n* = 125. **P = 0.009.

Activation of D1R has been consistently associated with pruning of neuronal extensions during early stages of development in vivo [41] and in vitro [43]. We have previously measured expression of D1R in MDNCs from healthy individuals [47]. Here we compare expression of D1R in MDNCs from CTL versus SCZ via flow cytometry. But first, we replicated previous reports indicating that monocytes do not express D1R [58] (Fig. 4A). In contrast, MDNCs from CTL and SCZ express D1R suggesting that our transdifferentiation protocol promotes its expression (Fig. 4B). Expression of D1R in MDNCs is not statistically different between CTL and patients with SCZ. However, if only medicated patients are included in the analysis, the difference becomes significant with patients expressing less D1R (Fig. 4C).

We then tested whether preincubation with SCH-23390, a D1R antagonist, prevents any of the structural changes elicited by dopamine 4 mM. SCH-23390 decreases pruning in LPN by 10% which is statistically significant. In contrast, pruning of LSN is not significantly affected by SCH-23390. No primary neurites were pruned either with dopamine or SCH-23390 + dopamine, while the number of secondary neurites and total number of neurites tends to decrease in the presence of SCH-23390 but without reaching statistical significance (Fig. 4D).

### Haloperidol effects on the structure and D1R expression of MDNCs

In order to determine whether antipsychotics impact levels of differentiation or the structure of MDNCs, we incubated monocytes from day 4 to 7 of the transdifferentiation process with haloperidol at known circulating levels (20 ng/ml) [59]. Haloperidol was selected because three patients with the highest differentiation efficiency were taking this antipsychotic. The incubation period consisting of 3 days was chosen to resemble in vivo conditions, where human monocytes remain in circulation for around 2 days [60].

**Fig. 5:**
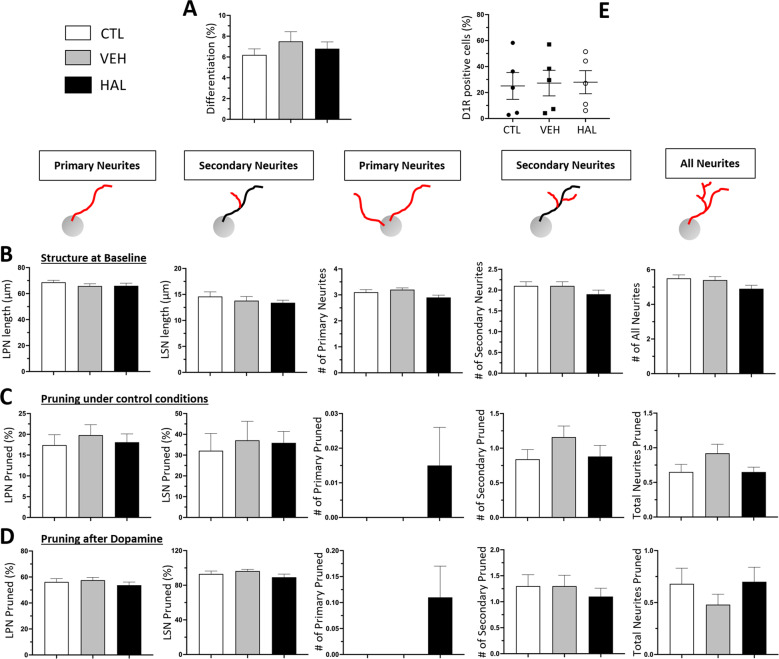
Haloperidol effects on the structure and D1R expression of MDNCs. **A** Bar graphs contrasting the differentiation percentage between cells treated with haloperidol (HAL), vehicle (VEH) or under control conditions (CTL). For CTL, 6.2 ± 0.58%; VEH, 7.5 ± 0.93%; HAL, 6.8 ± 0.65%; *P* = 0.69. The Kruskal–Wallis Test was used to make comparisons between groups. Data are given as mean ± SEM. Cells from 5 healthy subjects were included in the analysis. Cells characterized for CTL, *n* = 8048; VEH, *n* = 7152; HAL, *n* = 9300. **B** Bar graphs contrasting the structure of MDNCs at baseline after treatment from day 4 to 7 of the transdifferentiation process with haloperidol, vehicle or cells under control conditions. Structural parameters studied were: longest primary neurite (LPN), longest secondary neurite (LSN), number of primary neurites, number of secondary neurites and total number of neurites. The Kruskal–Wallis Test was used to make comparisons between groups. Data are given as mean ± SEM. Cells from 4 healthy subjects were included in the analysis. MDNCs traced for LSN for CTL, *n* = 247; VEH, *n* = 278; HAL, *n* = 305; for all other structural parameters; CTL, *n* = 339; VEH, *n* = 382; HAL, *n* = 431. **C** Bar graphs contrasting the amount of pruning evidenced in MDNCs after an hour of incubation on cells treated from day 4 to 7 of the transdifferentiation process with haloperidol, vehicle or cells under control conditions. The same structural parameter as in (**B**) were assessed. The Kruskal-Wallis Test was used to make comparisons between groups. Data are given as mean ± SEM. Cells from 3 healthy subjects were included in the analysis. MDNCs traced for LSN for CTL, *n* = 64; VEH, *n* = 85; HAL, *n* = 124; for all other structural parameters; CTL, *n* = 98; VEH, *n* = 113; HAL, *n* = 189. **D** Bar graphs contrasting the amount of pruning evidenced in MDNCs after an hour of incubation with dopamine 5 mM in cells treated from day 4 to 7 of the transdifferentiation process with haloperidol, vehicle or cells under control conditions. The same structural parameter as in (**B**) were assessed. The Kruskal–Wallis Test was used to make comparisons between groups. Data are given as mean ± SEM. Cells from 2 healthy subjects were included in the analysis. MDNCs traced for LSN for CTL, *n* = 28; VEH, *n* = 23; HAL, *n* = 37; for all other structural parameters; CTL, *n* = 57; VEH, *n* = 68; HAL, *n* = 81. **E** Dot plots contrasting expression of D1R in MDNCs after treatment from day 4 to 7 of the transdifferentiation process with HAL, VEH or cells under CTL conditions. For CTL, 25 ± 10.3%; VEH, 27.2 ± 9.8%; HAL, 27.9 ± 8.8%; *P* = 0.87. D1R expression was measured via flow cytometry. The Kruskal–Wallis Test was used to make comparisons between groups. Data are given as mean ± SEM. Cells from 5 healthy subjects were included in the analysis.

A non-parametric one-way ANOVA on the percentage of differentiated MDNCs established by phenotype, indicates CTL cells are not statistically different to those incubated with either vehicle (VEH) or haloperidol (HAL) (Fig. 5A). The structure of MDNCs also remains unchanged regardless of treatment or structural component studied (Fig. 5B & Supplementary Table S17).

We also analyzed whether haloperidol treatment impacts pruning. LPN from MDNCs cultured under CTL conditions, VEH or HAL retract equally. Likewise, the amount of retraction in LSN between the three culture conditions is similar. Similarly, the number of primary, secondary and total number of pruned neurites is comparable between the three groups (Fig. 5C & Supplementary Table S18).

The pruning effects of dopamine 5 mM on MDNCs was also investigated after haloperidol treatment from day 4 to 7. Pruning of LPN and LSN is equivalent between the three treatment conditions. The same is true for number of primary, secondary and total number of pruned neurites (Fig. 5D & Supplementary Table S19). Finally, we measured through flow cytometry whether haloperidol alters the expression of D1R. MCNCs’ expression of D1R is unaffected by HAL treatment (Fig. 5E).

## Discussion

Despite intense research during the last several decades, the pathophysiology of SCZ remains obscure. One of the main obstacles to study this mental illness is the difficulty of accessing neurons directly from patients. The extended lapse between SCZ putative origin during neurodevelopment and its clinical emergence makes this challenge even harder. In order to overcome this obstacle, we used MDNCs that allow us to test in vitro whether some of the very early neurodevelopmental steps involving the neuronal structure are deficient in SCZ. Particularly intriguing is the possibility that patients with SCZ carry an increased susceptibility to the pruning effects of dopamine. This susceptibility is likely to be more obvious in the early stages of neuronal development when dopamine pruning effects appear to be more prominent [40–46]. In fact, dopamine removes neuronal extensions even before commitment into any particular neuronal type is yet achieved [40, 42, 44, 45]. MDNCs stand within this neurodevelopmental window [47]. It is important to note that during the early stages of neurite formation neurites shorter than two times the soma size are more likely to retract spontaneously than longer extensions [61]. Thus, we studied only neurites longer than two times the soma size. In addition, we concentrated on analyzing the longest primary and secondary neurites as this approach limits excessive variation in neurite size, which could obscure subtle differences between patients and controls.

In a prior publication we showed that the structure of MDNCs is comparable to that of HDNs maintained in culture for 5 days [47]. As a further step to characterize the neurostructure of MDNCs, here we contrasted how MDNCs and HDNs respond to colchicine, a compound well-known in neuroscience for its ability to prune neuronal extensions [53]. MDNCs reproduce changes in LPN, number of primary neurites and total number of neurites found in HDNs (Fig. 1C). In contrast, MDNCs do not replicate variations in LSN or number of secondary neurites. These results suggest that findings from MDNCs involving pruning of LPN, number of primary neurites and/or total number of neurites are likely to be found in HDNs, while insights relating to secondary neurites would not.

Concerns have been raised about the reproducibility of results obtained with other cellular models [49, 50, 62]. Given these concerns, we have previously reported that MDNCs deliver reproducible results in a small cohort of healthy men [47]. Here we expanded our cohort to include women and we also increased the number of samples tested (Table 2). In our current cohort, reproducibility on the percentage of differentiated MDNCs is low, while the number of differentiated cells is consistent (Fig. 1A). Reproducibility in the percentage of differentiated cells is low not because of variations in the number of MDNCs as evidenced by our reproducibility results regarding number of differentiated cells (Fig. 1A). Instead, variations in the percentage of differentiated cells is driven by the number of undifferentiated cells. Results from several research teams indicate that there is a specific type of monocyte with pluripotent capacities [63–65] and our results suggest that the number of pluripotent monocytes remains constant with serial samples from healthy individuals while the amount of other cells can fluctuate.

We also tested the reproducibility of several neurostructural parameters and found different degrees of consistency (Fig. 1A). Results involving LPN were more consistent than those relating to LSN, number of secondary neurites and total number of neurites. However, for LSN, number of secondary neurites and total number of neurites only subject 6 delivered inconsistent results. Subject 6 appears to be an outlier as it is the only individual that conveyed inconsistent results in all parameters except for LPN. Reproducibility for number of primary neurites was low, found only in 60% of the subjects tested. Taking together the neurostructural comparison between MDNCs and HDNs (Fig. 1C) as well as the reproducibility of MDNCs (Fig. 1A), we conclude that differences between patients and controls that pertain to LPN and total number of neurites should be considered reliable. Results involving any of the other structural parameters studied, should be deemed less reliable, as they are either not found in HDNs or not consistently encountered in MDNCs.

After determining MDNCs’ reproducibility and its similarities with HDNs, we compared MDNCs from patients with SCZ versus cells from CTL. Patients were diagnosed using clinical interviews by experienced psychiatrists as well as medical records but no standardized scales were utilized to conclude patients had SCZ. Such approach should be considered as a limitation. We also included one patient with pervasive developmental disorder that presented psychotic symptoms. The cohort of patients and controls was matched by age and gender (Table 1). The number of PBMCs and monocytes did not differ between cohorts (Table 3). However, patients presented a small but statistically significant increase in the percentage of monocytes within PBMCs (Table 3) regardless on whether the patient with pervasive developmental disorder was included or excluded from the analysis (Supplementary Table S9). These results are consistent with several reports indicating patients with SCZ have higher numbers of monocytes [66]. The reason for this monocytosis might be related to some degree of inflammation present in patients with SCZ, as different publications have associated this psychotic disorder with plasmatic, transcriptomic and epigenetic signs of inflammation [67], including complement, coagulation factors [68], and lipidomic changes [69] but this possibility remains to be established.

While the path to differentiation is similar between groups (Fig. 2B & Supplementary Table S10), MDNCs from patients with SCZ differentiate more efficiently (Fig. 2C) and develop a more elaborated structure, presenting a higher number of primary neurites and longer secondary neurites (Fig. 2F). If the patient with pervasive developmental disorder is excluded from the analyses our findings remain the same (Supplementary Tables S11–S13). Previous publications using Induced Pluripotent Stem Cells (IPSCs) from patients with SCZ have also reported a more complex structure during early stages of neurodevelopment [70] though, not always [26].

In a previous publication we showed via genetic, immunofluorescence and flow cytometry that MDNCs decrease its expression of CD14 when compared to macrophages from the same individual [47]. A drop that was expected since CD14 is a marker for monocytes/macrophages. However, MDNCs from patients did not behave as anticipated. We observed a small decrease in the expression of CD14 that was not statistically significant (Fig. 2D). In contrast, MDNCs from CTL present a pronounced and statistically significant reduction in this marker for monocytes/macrophages (Fig. 2D). The structure of MDNCs from patients also behaves differently. While the neurostructure of MDNCs from CTL remains stable after the transdifferentiation process is completed, cells from patients continue to grow neurites during the following 24 hours (Fig. 2G & Supplementary Tables S14, S15). However, when the patient with pervasive developmental disorder is removed from the analyses this behavior is no longer significant (Supplementary Tables S16) which suggests MDNCs from patients with neurodevelopmental disorders other than SCZ may behave differently but this hypothesis requires further investigation.

After accounting in our statistical analysis for differences between SCZ and CTL in differentiation efficiency, size of secondary neurites and number of primary neurites, we challenged the structure of MDNCs’ with colchicine and dopamine. Both compounds are capable of pruning neuronal extensions through different mechanisms of action. Colchicine acts by directly depolymerizing microtubules [54] and thus, it is independent of membrane receptors, whereas dopamine relies on activation of D1R [41, 43]. The concentrations of colchicine and dopamine were selected based on its capacity to significantly increase pruning of LPN when compared to cells cultured under control conditions (Supplementary Fig. S1) while avoiding cellular damage. Also, because each tested concentration of colchicine and dopamine showed small increments on the degree of pruning when compared with lower concentrations of the same compound, even though, there were no statistically significant differences among the concentrations tested (Supplementary Fig. 1S). We expected subtle differences in the degree of pruning between patients and controls, therefore, we deduced that small increments in the level of pruning such as that observed with the different concentrations of colchicine and dopamine that we selected (Supplementary Fig. 1S) would reveal deficits present in patients with SCZ. Moreover, the three different concentrations of colchicine used are known to elicit pruning of extensions in neurons [55] and neuronal cell lines [56]. However, colchicine did not reveal any changes between MDNCs from SCZ and CTL (Fig. 3B–D). These results indicate that the microtubular component of the cytoskeleton is unaffected in MDNCs from patients with SCZ. Our data contrasts with previous reports suggesting patients with SCZ present deficits in microtubules organization and stability, granted those experiments were conducted using olfactory neuroepithelial cells [71, 72].

Two different concentrations of dopamine did not reveal differences in LPN, LSN, number of secondary neurites or total number of neurites between groups (Fig. 3E, F). Nonetheless, our experiments using SCH-23390, a specific D1R antagonist, indicate that at the concentration of dopamine 4 mM, this receptor is involved in pruning of LPN (Fig. 4D). Expression of D1R in MDNCs was similar in SCZ and CTL (Fig. 4C). When only medicated patients are included in the analysis, expression of D1R is significantly lower in patients with SCZ (Fig. 4C). It is therefore possible, that in medicated patients an increased susceptibility to the pruning effects of dopamine in LPN was masked by lower levels of D1R. MDNCs from medicated patients also evidenced increased pruning of primary neurites after exposure to the higher dose of dopamine (Fig. 3F). These results suggest that MDNCs from medicated patients are more susceptible to pruning of primary neurites and plausibly also to a partial removal of LPN by dopamine. It has to be kept in mind, however, that the reliability of data involving number of primary neurites from MDNCs is low because of the variability of results with serial samples (Fig. 1A). In contrast, results from LPN were consistent in every subject tested (Fig. 1A). While the potential contribution of antipsychotics in the pruning processes appears likely, several factors need to be pondered before drawing any conclusions.

Monocytes are exposed to medications during the 2–3 days they remain in blood circulation [60]. After monocyte extraction and while in culture for 20 days, there is no further exposure to medications. In fact, cell culture media is replaced in 4 occasions during the transdifferentiation process removing monocytes even further from their circulating environment [47]. Another important aspect to consider is that monocytes do not express dopamine receptors [58]. Here we replicated the absence of D1R expression in monocytes (Fig. 4A). Consequently, any antipsychotic influence would have to take place independently of dopamine receptors. Furthermore, incubations with haloperidol that mimic in vivo exposure, did not impact MDNCs’ differentiation, neurostructure or D1R expression (Fig. 5A–E). It is nonetheless possible, that antipsychotics could exert its effects via epigenetic mechanisms [73, 74]. There is indirect [75] and direct evidence [76] indicating that antipsychotics decrease dopaminergic signaling through epigenetic mechanisms. Antipsychotics can decrease the expression of dopamine receptors including D1R by increasing DNA methylation [76]. Therefore, our results showing decreased D1R expression in medicated patients (Fig. 4C) could be explained by the effects of antipsychotics. Lowering dopaminergic signaling is a mechanism that could protect against increased susceptibility to the pruning actions of dopamine. It is thus possible that antipsychotics could have prevented dopamine-elicited pruning in LPN through lowering D1R expression. With the information currently available, it seems possible that antipsychotics could have minimized rather than caused an increased susceptibility to dopamine pruning effects in medicated patients with SCZ. But further research is needed to establish the role of antipsychotics in dopamine-elicited pruning in MDNCs from patients with SCZ.

Another potential alternative to explain why we found increased susceptibility to dopamine pruning effects only in medicated patients could be patient heterogeneity. In this scenario, only a subset of patients with SCZ carries increased susceptibility to dopamine pruning actions. Further studies are needed to confirm this statement. In the meantime, the effects of medications on any of our comparisons between SCZ and CTL should be considered a confounding factor.

There is additional information that needs to be highlighted to properly appraise our results. While our cohort is among the largest in the field of SCZ and stem cells, only a subset of individuals were included when we tested MDNCs structural responses to colchicine and dopamine as described in Supplementary Table S1. Thus, the cohorts for some of these subset analyses are small (Supplementary Table S1). It is also important to consider that we used supra-physiological concentrations of dopamine to elicit rapid pruning of neuronal extensions. Consequently, our in vitro culture conditions do not resemble the microenvironment that surrounds neurons in vivo. Future studies using different concentrations of dopamine and longer incubation times are warranted as in vitro experiments using cells that carry the genetic susceptibility to SCZ and deliver results in only 20 days, open the possibility to a future cellular-based characterization of patients which is greatly needed in the psychiatric field.

In conclusion, our results suggest that a subset of patients with SCZ could carry an increased susceptibility to the pruning effects of dopamine during early stages of neurodevelopment but the possibility that antipsychotics, except for haloperidol, could have influenced our results through epigenetic mechanisms cannot be excluded.

## Supplementary information

Supplementary Table S1

Supplementary Tables S2 to S8

Supplementary Table S9

Supplementary Table S10

Supplementary Table S11

Supplementary Table S12

Supplementary Table S13

Supplementary Table S14

Supplementary Table S15

Supplementary Table S16

Supplementary Table S17

Supplementary Table S18

Supplementary Table S19

Supplementary Figure S1

Supplementary Figure Legend
