# Supplementary material for: Dopamine-induced pruning in monocyte-derived-neuronal-like cells (MDNCs) from patients with schizophrenia
Source: Mol Psychiatry. 2022 Apr 1;27(6):2787–802. doi: 10.1038/s41380-022-01514-w (PMC9156413; doi:10.1038/s41380-022-01514-w)
Supplement: Supplementary file 1 — Supplementary Table S1 [file 41380_2022_1514_MOESM1_ESM.docx]

**Supplementary Table S1.** Number, age range and gender of individuals included in each experiment.

| Experiment | Figure/Table | Number of individuals | Age  Range | Gender | Notes |
| --- | --- | --- | --- | --- | --- |
| Reproducibility of results with MDNCs | 1A  Table 2 | 8 | 25-78 | 5 males  3 females | Only one male belongs to the cohort described in Table 1. The rest were recruited for this experiment. |
| MDNCs used for comparison with HDNs under control conditions | 1C | 8 | 19-37 | 6 males  2 females | All individuals belong to the cohort described in Table 1 |
| MDNCs used for comparison with HDNs  after colchicine 0.5µM | 1C | 4 | 26-37 | 2 males  2 females | All individuals belong to the cohort described in Table 1 |
| Number of PBMCs and monocyte as well as percentage of monocytes  in controls | Tables  1 & 3 | 11* | 19-65 | 8 males**  3 females | All individuals belong to the cohort described in Table 1 |
| Number of PBMCs and monocyte as well as percentage of monocytes  in SCZ | Tables  1 & 3 | 13 | 19-67 | 11 males**  2 females | All individuals belong to the cohort described in Table 1 |
| Structural path to transdifferentiation days 4, 7 & 10 in controls | 2B | 12 | 19-65 | 9 males**  3 females | All individuals belong to the cohort described in Table 1 |
| Structural path to transdifferentiation days 4, 7 & 10 in SCZ | 2B | 13 | 19-67 | 11 males**  2 females | All individuals belong to the cohort described in Table 1 |
| Structural path to transdifferentiation day 13 in controls | 2B | 4 | 27-46 | 3 males**  1 female | All individuals belong to the cohort described in Table 1 |
| Structural path to transdifferentiation day 13 in SCZ | 2B | 10 | 19-67 | 9 males**  1 female | All individuals belong to the cohort described in Table 1 |
| Differentiation percentage  in controls | 2C | 12 | 19-65 | 9 males**  3 females | All individuals belong to the cohort described in Table 1 |
| Differentiation percentage  in SCZ | 2C | 13 | 19-67 | 11 males**  2 females | All individuals belong to the cohort described in Table 1 |
| Differentiation percentage  in MED^#^ | 2C | 11 | 19-67 | 9 males**  2 females | All individuals belong to the cohort described in Table 1 |
| Expression of CD14 in macrophages & MDNCs from controls | 2D | 9 | 26-65 | 7 males**  2 females | All individuals belong to the cohort described in Table 1 |
| Expression of CD14 in macrophages & MDNCs from SCZ | 2D | 11 | 19-67 | 9 males  2 females | All individuals belong to the cohort described in Table 1 |
| Expression of nestin in macrophages & MDNCs from controls | 2E | 2 | 37-46 | 2 males** | All individuals belong to the cohort described in Table 1 |
| Expression of nestin in macrophages & MDNCs from SCZ | 2E | 4 | 34-44 | 3 males  1 female | All individuals belong to the cohort described in Table 1 |
| MDNCs structural parameters at baseline in controls | 2F | 12 | 19-65 | 9 males**  3 females | All individuals belong to the cohort described in Table 1 |
| MDNCs structural parameters at baseline in SCZ | 2F | 13 | 19-67 | 11 males**  2 females | All individuals belong to the cohort described in Table 1 |
| MDNCs structural parameters at baseline in MED^#^ | 2F | 11 | 19-67 | 9 males**  2 females | All individuals belong to the cohort described in Table 1 |
| MDNCs structural parameters on day 20 vs. day 21 in controls | 2G | 7 | 19-37 | 4 males  3 females | All individuals belong to the cohort described in Table 1 |
| MDNCs structural parameters on day 20 vs. day 21 in SCZ | 2G | 11 | 19-67 | 9 males  2 females | All individuals belong to the cohort described in Table 1 |
| MDNCs structural parameters on day 20 vs. day 21 in MED^#^ | 2G | 10 | 19-67 | 8 males  2 females | All individuals belong to the cohort described in Table 1 |
| MDNCs structural responses after 1hr of culture under control conditions in controls | 3A | 8 | 19-37 | 6 males  2 females | All individuals belong to the cohort described in Table 1 |
| MDNCs structural responses after 1hr of culture under control conditions in SCZ | 3A | 10 | 19-67 | 9 males  1 female | All individuals belong to the cohort described in Table 1 |
| MDNCs structural responses after 1hr of culture under control conditions in MED^#^ | 3A | 8 | 19-67 | 7 males  1 female | All individuals belong to the cohort described in Table 1 |
| MDNCs structural responses after 1hr of colchicine 0.4µM in controls | 3B | 3 | 26-37 | 2 males  1 female | All individuals belong to the cohort described in Table 1 |
| MDNCs structural responses after 1hr of colchicine 0.4µM in SCZ | 3B | 7 | 19-44 | 7 males | All individuals belong to the cohort described in Table 1 |
| MDNCs structural responses after 1hr of colchicine 0.4µM in MED^#^ | 3B | 6 | 19-44 | 6 males | All individuals belong to the cohort described in Table 1 |
| MDNCs structural responses after 1hr of colchicine 0.5µM in controls | 3C | 4 | 26-37 | 2 males  2 females | All individuals belong to the cohort described in Table 1 |
| MDNCs structural responses after 1hr of colchicine 0.5µM in SCZ | 3C | 9 | 19-67 | 8 males  1 female | All individuals belong to the cohort described in Table 1 |
| MDNCs structural responses after 1hr of colchicine 0.5µM in MED^#^ | 3C | 8 | 19-67 | 7 males  1 female | All individuals belong to the cohort described in Table 1 |
| MDNCs structural responses after 1hr of colchicine 0.75µM in controls | 3D | 3 | 26-30 | 1 male  2 females | All individuals belong to the cohort described in Table 1 |
| MDNCs structural responses after 1hr of colchicine 0.75µM in SCZ | 3D | 7 | 19-67 | 6 males  1 female | All individuals belong to the cohort described in Table 1 |
| MDNCs structural responses after 1hr of colchicine 0.75µM in MED^#^ | 3D | 6 | 19-67 | 5 males  1 female | All individuals belong to the cohort described in Table 1 |
| MDNCs structural responses after 1hr of dopamine 4mM in controls | 3E | 6 | 19-37 | 4 males  2 females | All individuals belong to the cohort described in Table 1 |
| MDNCs structural responses after 1hr of dopamine 4mM in SCZ | 3E | 9 | 19-67 | 8 males  1 female | All individuals belong to the cohort described in Table 1 |
| MDNCs structural responses after 1hr of dopamine 4mM in MED^#^ | 3E | 7 | 19-67 | 6 males  1 female | All individuals belong to the cohort described in Table 1 |
| MDNCs structural responses after 1hr of dopamine 5mM in controls | 3F | 5 | 19-37 | 5 males | All individuals belong to the cohort described in Table 1 |
| MDNCs structural responses after 1hr of dopamine 5mM in SCZ | 3F | 7 | 25-44 | 6 males  1 female | All individuals belong to the cohort described in Table 1 |
| MDNCs structural responses after 1hr of dopamine 5mM in MED^#^ | 3F | 6 | 25-44 | 5 males  1 female | All individuals belong to the cohort described in Table 1 |
| Expression of D1R in MDNCs from controls | 4C | 7 | 19-37 | 7 males | All individuals belong to the cohort described in Table 1 |
| Expression of D1R in MDNCs from SCZ | 4C | 7 | 26-44 | 5 males  2 females | All individuals belong to the cohort described in Table 1 |
| Expression of D1R in MDNCs from MED^#^ | 4C | 5 | 26-44 | 3 males  2 females | All individuals belong to the cohort described in Table 1 |
| Role of D1R in dopamine-elicited pruning in MDNCs | 4D | 3 | 19-29 | 3 males | Two controls and one unmedicated patient with SCZ. All individuals belong to the cohort described in Table 1 |
| Differentiation percentage after treatment with haloperidol, vehicle or control | 5A | 5 | 23-59 | 3 males  2 females | Only one male belongs to the cohort described in Table 1. The rest were recruited for this experiment. |
| MDNCs structure at baseline after treatment with haloperidol, vehicle or control | 5B | 4 | 38-59 | 3 males  1 female | Only one male belongs to the cohort described in Table 1. The rest were recruited for this experiment. |
| Pruning of MDNCs’ extensions after treatment with haloperidol, vehicle or control | 5C | 3 | 38-59 | 2 males  1 female | All individuals were recruited for this experiment. |
| Dopamine-elicited pruning in MDNCs after treatment with haloperidol, vehicle or control | 5D | 2 | 23-59 | 2 females | All individuals were recruited for this experiment. |
| Determination of colchicine concentration for control conditions | Fig. S1A | 8 | 19-37 | 6 males  2 females | All individuals belong to the cohort described in Table 1 |
| Determination of colchicine concentration for colchicine 0.4µM | Fig. S1A | 4 | 26-37 | 2 males  2 females | All individuals belong to the cohort described in Table 1 |
| Determination of colchicine concentration for colchicine 0.5µM | Fig. S1A | 4 | 26-37 | 2 males  2 females | All individuals belong to the cohort described in Table 1 |
| Determination of colchicine concentration for colchicine 0.75µM | Fig. S1A | 3 | 26-30 | 1 male  2 females | All individuals belong to the cohort described in Table 1 |
| Determination of colchicine concentration for control conditions | Fig. S1B | 8 | 19-37 | 6 males  2 females | All individuals belong to the cohort described in Table 1 |
| Determination of colchicine concentration for dopamine 4mM | Fig. S1B | 6 | 19-37 | 4 males  2 females | All individuals belong to the cohort described in Table 1 |
| Determination of colchicine concentration for dopamine 5mM | Fig. S1B | 5 | 19-37 | 5 males | All individuals belong to the cohort described in Table 1 |

*We did not have access to the number of PBMCs & monocytes for one control subject. **One individual was tested twice.

^#^Only medicated patients.

^*^Some of the cells obtained from a single donor were used for more than one type of experiment, ^**^Supplementary, ^***^monocyte-derived-neuronal-like cells, ^****^Glutamic acid decarboxylase.
