# Supplementary material for: Dopamine-induced pruning in monocyte-derived-neuronal-like cells (MDNCs) from patients with schizophrenia
Source: Mol Psychiatry. 2022 Apr 1;27(6):2787–802. doi: 10.1038/s41380-022-01514-w (PMC9156413; doi:10.1038/s41380-022-01514-w)
Supplement: Supplementary file 2 — Supplementary Tables S2 to S8 [file 41380_2022_1514_MOESM2_ESM.docx]

**Supplementary Table S2.** Reproducibility of MDNCs’ differentiation percentage after serial samples from healthy individuals.

| Differentiation Percentage | | | |
| --- | --- | --- | --- |
| Subject | Difference  Mean | 95% CL range | Satterthwaite  test |
| S1 | -0.031 | -0.061 to -0.0009 | *P* = 0.04 |
| S2 | 0.040 | 0.015 to 0.065 | *P* = 0.003 |
| S3 | -0.090 | -0.145 to -0.036 | *P* = 0.002 |
| S4 | -0.014 | -0.047 to 0.020 | *P* = 0.38 |
| S5 | -0.047 | -0.102 to 0.008 | *P* = 0.08 |
| S6 | -0.042 | -0.072 to -0.012 | *P* = 0.007 |
| S7 | 0.012 | -0.042 to 0.066 | *P* = 0.63 |
| S8 | 0.013 | -0.001 to 0.027 | *P* = 0.07 |

CL = confidence interval

**Supplementary Table S3.** Reproducibility in number of differentiated MDNCs after serial samples from healthy individuals.

| Number of differentiated MDNCs | | | |
| --- | --- | --- | --- |
| Subject | Difference  Mean | 95% CL range | Satterthwaite  test |
| S1 | 0.312 | -0.508 to 1.132 | *P* = 0.43 |
| S2 | 0.461 | -0.054 to 0.976 | *P* = 0.07 |
| S3 | 0.244 | -0.264 to 0.752 | *P* = 0.32 |
| S4 | 0.333 | -0.946 to 1.613 | *P* = 0.58 |
| S5 | 0.577 | -0.418 to 1.572 | *P* = 0.23 |
| S6 | 0.741 | 0.220 to 1.262 | *P* = 0.006 |
| S7 | 0.566 | -0.525 to 1.656 | *P* = 0.28 |
| S8 | -0.351 | -0.899 to 0.196 | *P* = 0.20 |

CL = confidence interval

**Supplementary Table S4.** Reproducibility in longest primary neurite (LPN) from MDNCs after serial samples from healthy individuals.

| Longest Primary Neurite | | | |
| --- | --- | --- | --- |
| Subject | Difference  Mean | 95% CL range | Satterthwaite  test |
| S1 | 0.33 | -17.36 to 16.69 | *P* = 0.96 |
| S2 | 1.94 | -23.97 to 27.85 | *P* = 0.87 |
| S3 | 5.4 | -21.48 to 32.3 | *P* = 0.68 |
| S4 | 1.79 | -14.58 to 18.17 | *P* = 0.82 |
| S5 | -9.27 | -25.27 to 6.72 | *P* = 0.24 |
| S6 | -3.88 | -17.54 to 9.77 | *P* = 0.57 |
| S7 | 2.56 | -16.53 to 21.65 | *P* = 0.78 |
| S8 | 5.01 | -7.16 to 17.19 | *P* = 0.41 |

CL = confidence interval

**Supplementary Table S5.** Reproducibility in longest secondary neurite (LSN) from MDNCs after serial samples from healthy individuals.

| Longest Secondary Neurite | | | |
| --- | --- | --- | --- |
| Subject | Difference  Mean | 95% CL range | Satterthwaite  test |
| S1 | -1.61 | -6.38 to 3.15 | *P* = 0.50 |
| S2 | -1.01 | -6.79 to 4.75 | *P* = 0.72 |
| S3 | 4.33 | -1.72 to 10.4 | *P* = 0.15 |
| S4 | -1.65 | -6.94 to 3.93 | *P* = 0.53 |
| S5 | 4.65 | -2.33 to 11.63 | *P* = 0.18 |
| S6 | -4.26 | -8.48 to -0.054 | *P* = 0.04 |
| S7 | -5.19 | -11.97 to 1.58 | *P* = 0.13 |
| S8 | 0.70 | -4.68 to 6.08 | *P* = 0.79 |

CL = confidence interval

**Supplementary Table S6.** Reproducibility in number of primary neurites from MDNCs after serial samples from healthy individuals.

| Number of Primary Neurites | | | |
| --- | --- | --- | --- |
| Subject | Difference  Mean | 95% CL range | Satterthwaite  test |
| S1 | 1.9 | 1.02 to 2.79 | *P* = 0.0001 |
| S2 | 1.82 | 0.62 to 3.02 | *P* = 0.005 |
| S3 | 0.12 | -0.9 to 1.14 | *P* = 0.81 |
| S4 | 0.29 | -0.53 to 1.12 | *P* = 0.47 |
| S5 | 0.06 | -1.16 to 1.28 | *P* = 0.91 |
| S6 | -1.07 | -1.74 to 0.4 | *P* = 0.002 |
| S7 | 0.26 | -0.88 to 1.42 | *P* = 0.64 |
| S8 | -0.34 | -1.13 to 0.44 | *P* = 0.38 |

CL = confidence interval

**Supplementary Table S7.** Reproducibility in number of secondary neurites from MDNCs after serial samples from healthy individuals.

| Number of Secondary Neurites | | | |
| --- | --- | --- | --- |
| Subject | Difference  Mean | 95% CL range | Satterthwaite  test |
| S1 | 0.088 | -2.4 to 2.58 | *P* = 0.94 |
| S2 | -0.27 | -4.14 to 3.6 | *P* = 0.88 |
| S3 | -0.41 | -3.36 to 2.53 | *P* = 0.77 |
| S4 | -1.28 | -2.94 to 0.38 | *P* = 0.12 |
| S5 | 0.58 | -3.43 to 4.59 | *P* = 0.77 |
| S6 | -3.57 | -5.69 to 1.46 | *P* = 0.001 |
| S7 | -0.75 | -3.98 to 2.48 | *P* = 0.64 |
| S8 | -0.92 | -2.91 to 1.06 | *P* = 0.35 |

CL = confidence interval

**Supplementary Table S8.** Reproducibility in total number of neurites from MDNCs after serial samples from healthy individuals.

| Total Number of Neurites | | | |
| --- | --- | --- | --- |
| Subject | Difference  Mean | 95% CL range | Satterthwaite  test |
| S1 | 2.51 | -0.89 to 5.91 | *P* = 0.14 |
| S2 | 1.0 | -4.42 to 6.45 | *P* = 0.70 |
| S3 | 0.54 | -3.08 to 4.16 | *P* = 0.76 |
| S4 | -1.55 | -4.19 to 1.09 | *P* = 0.24 |
| S5 | 0.77 | -4.89 to 6.44 | *P* = 0.78 |
| S6 | -5.88 | -8.91 to -2.85 | *P* = 0.0002 |
| S7 | -1.21 | -5.93 to 3.5 | *P* = 0.60 |
| S8 | -1.05 | -4.02 to 1.91 | *P* = 0.48 |

CL = confidence interval
