# Supplementary material for: Dopamine-induced pruning in monocyte-derived-neuronal-like cells (MDNCs) from patients with schizophrenia
Source: Mol Psychiatry. 2022 Apr 1;27(6):2787–802. doi: 10.1038/s41380-022-01514-w (PMC9156413; doi:10.1038/s41380-022-01514-w)
Supplement: Supplementary file 3 — Supplementary Table S9 [file 41380_2022_1514_MOESM3_ESM.docx]

**Supplementary Table S9.** Gender, age and number of monocytes and peripheral blood mononuclear cells at baseline from controls versus patients excluding a patient with pervasive developmental disorder.

|  | **Controls**  **(*n* = 13)** | **Schizophrenia**  **(*n* = 13)** | ***P* value** |
| --- | --- | --- | --- |
| **Sex** | 77% men | 84% men | *P* = 1.0 |
| **Age**  **(mean ± SEM)**  **(range)** | 32.6 ± 3.2  (19-65 years) | 34.3 ± 3.5  (19-67 years) | *P* = 0.71 |
| **Number of**  **Monocytes**  **(mean ± SEM)**  **(range)** | 6.58 ± 0.72  (3.8-12.3 million) | 8.2 ± 1.25  (2-17 million) | *P* = 0.28 |
| **Number of**  ***PBMCs**  **(mean ± SEM)**  **(range)** | 71.65 ± 6.06  (43-101.5 million) | 59.46 ± 4.06  (39-88 million) | *P* = 0.10 |
| **Percentage of**  **Monocytes**  **(mean ± SEM)**  **(range)** | 9.6 ± 0.64%  (6.3-13.1%) | 13.1 ± 1.4%  (5-21.7%) | *P* = 0.04 |

*PBMCs = Peripheral blood mononuclear cells.
