# Supplementary material for: Dopamine-induced pruning in monocyte-derived-neuronal-like cells (MDNCs) from patients with schizophrenia
Source: Mol Psychiatry. 2022 Apr 1;27(6):2787–802. doi: 10.1038/s41380-022-01514-w (PMC9156413; doi:10.1038/s41380-022-01514-w)
Supplement: Supplementary file 4 — Supplementary Table S10 [file 41380_2022_1514_MOESM4_ESM.docx]

**Supplementary Table S10.** Structural path to transdifferentiation in patients versus controls.

| Day | Structural  Stage | Controls | Patients with  schizophrenia | ANOVAs |
| --- | --- | --- | --- | --- |
| Day 4 | RC | 32.3 ± 4.6% | 33.3 ± 4.4% | *P* = 0.88 |
|  | SM | 10.5 ± 2.3% | 11.6 ± 2.2% | *P* = 0.72 |
|  | FS | 29.9 ± 5% | 34 ± 4.9% | *P* = 0.56 |
|  | UC | 27.1 ± 2.9% | 20.9 ± 2.8% | *P* = 0.14 |
| Day 7 | RC | 22 ± 4.8% | 13.7 ± 4.6% | *P* = 0.22 |
|  | SM | 23.2 ± 5.7% | 26.4 ± 5.5% | *P* = 0.69 |
|  | FS | 32.6 ± 5.1% | 44.2 ± 4.9% | *P* = 0.11 |
|  | UC | 21.9 ± 1.9% | 15.5 ± 1.8% | *P* = 0.02 |
| Day 10 | RC | 14.8 ± 4% | 12.2 ± 3.9% | *P* = 0.64 |
|  | SM | 36.6 ± 7.1% | 34.4 ± 6.9% | *P* = 0.82 |
|  | FS | 28.1 ± 5.5% | 35.1 ± 5.3% | *P* =0.36 |
|  | UC | 20.3 ± 2.4% | 18.1 ± 2.4% | *P* = 0.52 |
| Day 13 | RC | 13.5 ± 3.5% | 10.4 ± 2.4% | *P* = 0.47 |
|  | SM | 29.9 ± 6.5% | 37.6 ± 4.4% | *P* = 0.19 |
|  | FS | 41 ± 7.1% | 27.9 ± 4.8% | *P* = 0.15 |
|  | UC | 18.3 ± 3.1% | 23.9 ± 2% | *P* = 0.15 |

RC = rounded cell, SM = standard macrophage, FS = fibroblastic shape and

UC = uncharacterized cells.
