# Supplementary material for: Dopamine-induced pruning in monocyte-derived-neuronal-like cells (MDNCs) from patients with schizophrenia
Source: Mol Psychiatry. 2022 Apr 1;27(6):2787–802. doi: 10.1038/s41380-022-01514-w (PMC9156413; doi:10.1038/s41380-022-01514-w)
Supplement: Supplementary file 5 — Supplementary Table S11 [file 41380_2022_1514_MOESM5_ESM.docx]

**Supplementary Table S11.** Structural path to transdifferentiation in controls versus patients excluding one patient with pervasive developmental disorder.

| Day | Structural  Stage | Controls | Patients with  schizophrenia | ANOVAs |
| --- | --- | --- | --- | --- |
| Day 4 | RC | 32.3 ± 4.6% | 34.5 ± 6.4% | *P* = 0.73 |
|  | SM | 10.5 ± 2.3% | 12.0 ± 3.2% | *P* = 0.64 |
|  | FS | 29.9 ± 5% | 32.7 ± 7.2% | *P* = 0.70 |
|  | UC | 27.1 ± 2.9% | 20.5 ± 4.2% | *P* = 0.13 |
| Day 7 | RC | 22 ± 4.8% | 12.6 ± 6.8% | *P* = 0.17 |
|  | SM | 23.2 ± 5.7% | 27.0 ± 8.2% | *P* = 0.65 |
|  | FS | 32.6 ± 5.1% | 45.3 ± 7.3% | *P* = 0.09 |
|  | UC | 21.9 ± 1.9% | 15.0 ± 2.7% | *P* = 0.01 |
| Day 10 | RC | 14.8 ± 4% | 11.9 ± 5.8% | *P* = 0.61 |
|  | SM | 36.6 ± 7.1% | 35.6 ± 10.2% | *P* = 0.92 |
|  | FS | 28.1 ± 5.5% | 34.4 ± 7.9% | *P* =0.43 |
|  | UC | 20.3 ± 2.4% | 18.0 ± 3.6% | *P* = 0.51 |
| Day 13 | RC | 13.5 ± 3.5% | 10.2 ± 4.5% | *P* = 0.48 |
|  | SM | 29.9 ± 6.5% | 38.3 ± 8.3% | *P* = 0.19 |
|  | FS | 41 ± 7.1% | 27.1 ± 8.9% | *P* = 0.14 |
|  | UC | 18.3 ± 3.1% | 24.2 ± 3.9% | *P* = 0.15 |

RC = rounded cell, SM = standard macrophage, FS = fibroblastic shape and

UC = uncharacterized cells.
