# Supplementary material for: Dopamine-induced pruning in monocyte-derived-neuronal-like cells (MDNCs) from patients with schizophrenia
Source: Mol Psychiatry. 2022 Apr 1;27(6):2787–802. doi: 10.1038/s41380-022-01514-w (PMC9156413; doi:10.1038/s41380-022-01514-w)
Supplement: Supplementary file 6 — Supplementary Table S12 [file 41380_2022_1514_MOESM6_ESM.docx]

**Supplementary Table S12.** Structural differences at baseline between MDNCs from controls (CTL) versus patients with schizophrenia (SCZ) excluding one individual with pervasive developmental disorder.

| Structural  component | CTL  *N*=13 | SCZ*  *N*=13 | *P*  value |
| --- | --- | --- | --- |
| LPN (µm) | 91.1 ± 2.6 | 94.1 ± 3.7 | 0.42 |
| LSN (µm) | 15.8 ± 0.73 | 18.3 ± 1.0 | 0.02 |
| # of Primaries | 4.3 ± 0.09 | 4.6 ± 0.13 | 0.04 |
| # of Secondaries | 5.1 ± 0.52 | 6.2 ± 0.74 | 0.16 |
| # of all neurites | 8.8 ± 0.64 | 10.3 ± 0.91 | 0.11 |

*Excluding one patient with pervasive developmental disorder.

LPN=longest primary neurite, LSN=longest secondary neurite.
