# Supplementary material for: Dopamine-induced pruning in monocyte-derived-neuronal-like cells (MDNCs) from patients with schizophrenia
Source: Mol Psychiatry. 2022 Apr 1;27(6):2787–802. doi: 10.1038/s41380-022-01514-w (PMC9156413; doi:10.1038/s41380-022-01514-w)
Supplement: Supplementary file 7 — Supplementary Table S13 [file 41380_2022_1514_MOESM7_ESM.docx]

**Supplementary Table S13.** Structural differences at baseline between MDNCs from controls (CTL) versus only medicated patients with schizophrenia (MED) excluding one individual with pervasive developmental disorder.

| Structural  component | CTL  *N*=13 | MED*  *N*=11 | *P*  value |
| --- | --- | --- | --- |
| LPN (µm) | 91.1 ± 2.6 | 95.3 ± 3.8 | 0.28 |
| LSN (µm) | 15.8 ± 0.73 | 18.0 ± 1.1 | 0.06 |
| # of Primaries | 4.3 ± 0.09 | 4.7 ± 0.13 | 0.01 |
| # of Secondaries | 5.1 ± 0.52 | 6.3 ± 0.80 | 0.18 |
| # of all neurites | 8.8 ± 0.64 | 10.4 ± 0.99 | 0.13 |

*Excluding one patient with pervasive developmental disorder.

LPN=longest primary neurite, LSN=longest secondary neurite.
