# Supplementary material for: Dopamine-induced pruning in monocyte-derived-neuronal-like cells (MDNCs) from patients with schizophrenia
Source: Mol Psychiatry. 2022 Apr 1;27(6):2787–802. doi: 10.1038/s41380-022-01514-w (PMC9156413; doi:10.1038/s41380-022-01514-w)
Supplement: Supplementary file 8 — Supplementary Table S14 [file 41380_2022_1514_MOESM8_ESM.docx]

**Supplementary Table S14.** Structural differences between day 20 and day 21 in MDNCs from control individuals (CTL).

| Structural  component | CTL  Day 20 | CTL  Day 21 | *P*  value |
| --- | --- | --- | --- |
| LPN (µm) | 92.0 ± 3.6 | 95.3 ± 3.6 | 0.23 |
| LSN (µm) | 16.4 ± 1.3 | 15.3 ± 1.3 | 0.25 |
| # of Primaries | 4.2 ± 0.07 | 4.3 ± 0.07 | 0.20 |
| # of Secondaries | 4.9 ± 0.5 | 4.3 ± 0.5 | 0.16 |
| # of all neurites | 8.4 ± 0.6 | 7.9 ± 0.6 | 0.29 |

LPN=longest primary neurite, LSN=longest secondary neurite.
