# Supplementary material for: Dopamine-induced pruning in monocyte-derived-neuronal-like cells (MDNCs) from patients with schizophrenia
Source: Mol Psychiatry. 2022 Apr 1;27(6):2787–802. doi: 10.1038/s41380-022-01514-w (PMC9156413; doi:10.1038/s41380-022-01514-w)
Supplement: Supplementary file 9 — Supplementary Table S15 [file 41380_2022_1514_MOESM9_ESM.docx]

**Supplementary Table S15.** Structural differences between day 20 and day 21 in MDNCs from patients with schizophrenia (SCZ) and only medicated patients with schizophrenia (MED).

| Structural  component | SCZ  Day 20 | SCZ  Day 21 | *P*  value | MED  Day 20 | MED  Day 21 | *P*  value |
| --- | --- | --- | --- | --- | --- | --- |
| LPN (µm) | 93.7 ± 2.9 | 96.2 ± 2.9 | 0.08 | 94.1 ± 3.2 | 96.1 ± 3.2 | 0.15 |
| LSN (µm) | 18.7 ± 0.75 | 19.1 ± 0.74 | 0.6 | 18.6 ± 0.8 | 18.9 ± 0.7 | 0.71 |
| # of Primaries | 4.63 ± 0.13 | 4.67 ± 0.13 | 0.67 | 4.60 ± 0.14 | 4.68 ± 0.14 | 0.85 |
| # of Secondaries | 6.0 ± 0.4 | 6.6 ± 0.4 | 0.10 | 5.9 ± 0.48 | 6.5 ± 0.47 | 0.14 |
| # of all neurites | 9.9 ± 0.5 | 10.8 ± 0.5 | 0.02 | 9.9 ± 0.61 | 10.8 ± 0.61 | 0.04 |

LPN=longest primary neurite, LSN=longest secondary neurite.
