# Supplementary material for: Dopamine-induced pruning in monocyte-derived-neuronal-like cells (MDNCs) from patients with schizophrenia
Source: Mol Psychiatry. 2022 Apr 1;27(6):2787–802. doi: 10.1038/s41380-022-01514-w (PMC9156413; doi:10.1038/s41380-022-01514-w)
Supplement: Supplementary file 10 — Supplementary Table S16 [file 41380_2022_1514_MOESM10_ESM.docx]

**Supplementary Table S16.** Structural differences between day 20 and day 21 in MDNCs from patients with schizophrenia (SCZ) and only medicated patients with schizophrenia (MED) excluding one individual with pervasive developmental disorder from both cohorts.

| Structural  component | SCZ  Day 20 | SCZ  Day 21 | *P*  value | MED  Day 20 | MED  Day 21 | *P*  value |
| --- | --- | --- | --- | --- | --- | --- |
| LPN (µm) | 94.0 ± 3.0 | 97.0 ± 1.3 | 0.16 | 94.5 ± 3.4 | 96.8 ± 1.3 | 0.31 |
| LSN (µm) | 18.6 ± 0.73 | 18.5 ± 0.65 | 0.97 | 18.5 ± 0.77 | 18.3 ± 0.70 | 0.80 |
| # of Primaries | 4.5 ± 0.14 | 4.7 ± 0.09 | 0.35 | 4.5 ± 0.15 | 4.7 ± 0.09 | 0.49 |
| # of Secondaries | 6.1 ± 0.46 | 6.5 ± 0.32 | 0.20 | 6.0 ± 0.51 | 6.4 ± 0.36 | 0.29 |
| # of all neurites | 10.1 ± 0.60 | 10.7 ± 0.34 | 0.06 | 10.0 ± 0.67 | 10.7 ± 0.38 | 0.09 |

LPN=longest primary neurite, LSN=longest secondary neurite.
