# Supplementary material for: Dopamine-induced pruning in monocyte-derived-neuronal-like cells (MDNCs) from patients with schizophrenia
Source: Mol Psychiatry. 2022 Apr 1;27(6):2787–802. doi: 10.1038/s41380-022-01514-w (PMC9156413; doi:10.1038/s41380-022-01514-w)
Supplement: Supplementary file 11 — Supplementary Table S17 [file 41380_2022_1514_MOESM11_ESM.docx]

**Supplementary Table S17.** Structural differences at baseline between MDNCs incubated under control conditions (CTL), with vehicle (VEH) or with haloperidol (HAL).

| Structural  component | CTL | VEH | HAL | *P*  value |
| --- | --- | --- | --- | --- |
| LPN (µm) | 68.7 ± 1.5 | 65.8 ± 1.8 | 66 ± 2.0 | 0.52 |
| LSN (µm) | 14.6 ± 0.9 | 13.8 ± 0.8 | 13.4 ± 0.5 | 0.65 |
| # of Primaries | 3.1 ± 0.1 | 3.2 ± 0.07 | 2.9 ± 0.09 | 0.08 |
| # of Secondaries | 2.1 ± 0.1 | 2.1 ± 0.1 | 1.9 ± 0.1 | 0.59 |
| # of all neurites | 5.5 ± 0.2 | 5.4 ± 0.2 | 4.9 ± 0.2 | 0.27 |

LPN=longest primary neurite, LSN=longest secondary neurite.
