# Supplementary material for: Dopamine-induced pruning in monocyte-derived-neuronal-like cells (MDNCs) from patients with schizophrenia
Source: Mol Psychiatry. 2022 Apr 1;27(6):2787–802. doi: 10.1038/s41380-022-01514-w (PMC9156413; doi:10.1038/s41380-022-01514-w)
Supplement: Supplementary file 12 — Supplementary Table S18 [file 41380_2022_1514_MOESM12_ESM.docx]

**Supplementary Table S18.** Pruning in MDNCs incubated under control conditions (CTL), with vehicle (VEH) or with haloperidol (HAL).

| Structural  component | CTL | VEH | HAL | *P*  value |
| --- | --- | --- | --- | --- |
| LPN (%) | 17.4 ± 2.5 | 19.8 ± 2.5 | 18.1 ± 2.0 | 0.92 |
| LSN (%) | 32.1 ± 8.3 | 37.1 ± 9.2 | 35.9 ± 5.5 | 0.48 |
| # of Primaries | 0 ± 0 | 0 ± 0 | 0.015 ± 0.011 | 0.98 |
| # of Secondaries | 0.84 ± 0.14 | 1.16 ± 0.16 | 0.88 ± 0.16 | 0.33 |
| # of all neurites | 0.65 ± 0.11 | 0.92 ± 0.13 | 0.65 ± 0.07 | 0.24 |

LPN=longest primary neurite, LSN=longest secondary neurite.
