# Supplementary material for: Dopamine-induced pruning in monocyte-derived-neuronal-like cells (MDNCs) from patients with schizophrenia
Source: Mol Psychiatry. 2022 Apr 1;27(6):2787–802. doi: 10.1038/s41380-022-01514-w (PMC9156413; doi:10.1038/s41380-022-01514-w)
Supplement: Supplementary file 13 — Supplementary Table S19 [file 41380_2022_1514_MOESM13_ESM.docx]

**Supplementary Table S19.** Pruning after treatment with dopamine 5mM in MDNCs incubated under control conditions (CTL), with vehicle (VEH) or with haloperidol (HAL).

| Structural  component | CTL | VEH | HAL | *P*  value |
| --- | --- | --- | --- | --- |
| LPN (%) | 56.2 ± 2.5 | 57.5 ± 2.2 | 53.7 ± 2.4 | 0.54 |
| LSN (%) | 93 ± 3.4 | 96.4 ± 2 | 89.2 ± 3.6 | 0.75 |
| # of Primaries | 0 ± 0 | 0 ± 0 | 0.11 ± 0.06 | 0.83 |
| # of Secondaries | 1.3 ± 0.22 | 1.3 ± 0.21 | 1.1 ± 0.16 | 0.79 |
| # of all neurites | 0.68 ± 0.15 | 0.48 ± 0.1 | 0.7 ± 0.14 | 0.54 |

LPN=longest primary neurite, LSN=longest secondary neurite.
