# Supplementary figures and images for: Dopamine-induced pruning in monocyte-derived-neuronal-like cells (MDNCs) from patients with schizophrenia
Source: Mol Psychiatry. 2022 Apr 1;27(6):2787–802. doi: 10.1038/s41380-022-01514-w (PMC9156413; doi:10.1038/s41380-022-01514-w)

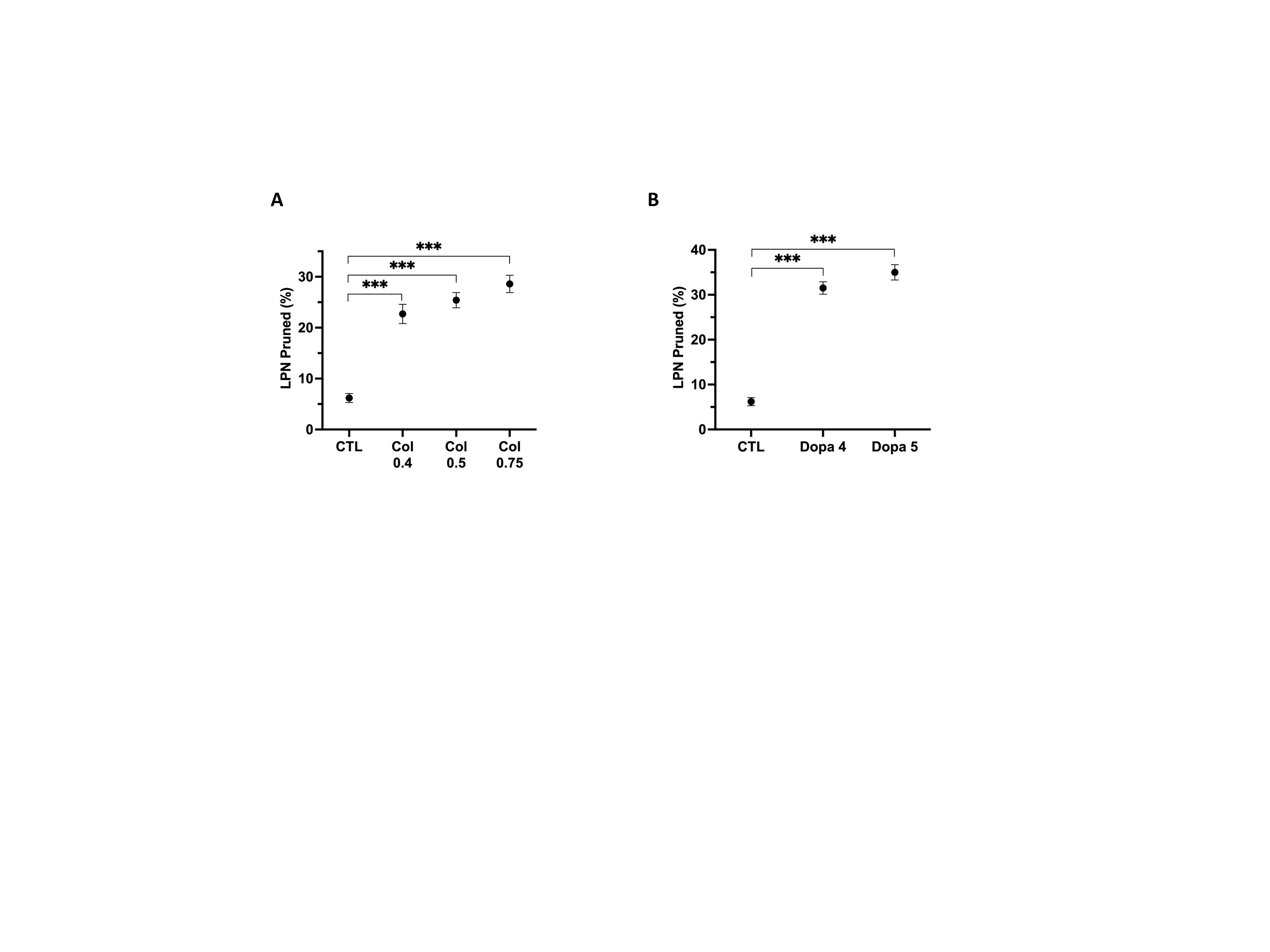

Supplement: Supplementary file 14 — Supplementary Figure S1 [file 41380_2022_1514_MOESM14_ESM.tif]
