# Supplementary material for: Dopamine-induced pruning in monocyte-derived-neuronal-like cells (MDNCs) from patients with schizophrenia
Source: Mol Psychiatry. 2022 Apr 1;27(6):2787–802. doi: 10.1038/s41380-022-01514-w (PMC9156413; doi:10.1038/s41380-022-01514-w)
Supplement: Supplementary file 15 — Supplementary Figure Legend [file 41380_2022_1514_MOESM15_ESM.docx]

**Supplementary Figure Legend**

**Supplementary Figure S1. Pruning of longest primary neurite (LPN) from MDNCs after treatment with different concentrations of colchicine or dopamine. (A)** Dot plots contrasting pruning of LPN from MDNCs after one hour under controlled culture conditions versus one hour of treatment with either colchicine (Col) 0.4, 0.5 or 0.75µM. One-way ANOVA indicated significant differences between groups [F(3, 1605) = 63.3, *P* < 0.00001]. Bonferroni correction indicates differences are driven by comparisons between control conditions (CTL) and the three different concentrations of colchicine (CTL, 6.2 ± 0.9%; Col 0.4µM, 22.7 ± 1.9%; *P* < 0.00001; Col 0.5µM, 25.4 ± 1.5%; *P* < 0.00001; Col 0.75µM, 28.6 ± 1.7%; *P* < 0.00001). After Bonferroni correction, no significant differences remained when comparing pruning of LPN among the three concentrations of colchicine tested (Col 0.4µM, 22.7 ± 1.9%; Col 0.5µM, 25.4 ± 1.5%; *P* = 0.28; Col 0.75µM, 28.6 ± 1.7%; *P* = 0.02) ( Col 0.5µM, 25.4 ± 1.5%; Col 0.75µM, 28.6 ± 1.7%; *P* = 0.016). Data are given as mean ± SEM. The number of cells included in the analysis were; for control conditions, *n* = 656 MDNCs from 8 controls, for colchicine 0.4µM, *n* = 255 MDNCs from 4 controls, for colchicine 0.5µM, *n* = 401 MDNCs from 4 controls and for colchicine 0.75µM, *n* = 297 MDNCs from 3 controls. **(B)** Dot plots contrasting pruning of LPN from MDNCs after one hour under controlled culture conditions versus one hour of treatment with either dopamine (Dopa) 4mM or dopamine 5mM. One-way ANOVA indicated significant differences between groups [F(2, 1474) = 160.5, *P* < 0.00001]. Bonferroni correction indicates differences are driven by comparisons between CTL and the two different concentrations of dopamine (CTL, 6.2 ± 0.9%; Dopa 4mM, 31.5 ± 1.4%; *P* < 0.00001; Dopa 5mM, 35.0 ± 1.7%; *P* < 0.00001). There are no statistical differences in pruning of LPN when comparing the two concentrations of dopamine (Dopa 4mM, 31.5 ± 1.4%; Dopa 5mM, 35.0 ± 1.7%; *P* = 0.12). Data are given as mean ± SEM. The number of cells included in the analysis were; for control conditions, *n* = 656 MDNCs from 8 controls, for dopamine 4mM, *n* = 477 MDNCs from 6 controls and for dopamine 5mM, *n* = 344 MDNCs from 5 controls. ****P* < 0.00001.
